# Structural characterization of anti-CCL5 activity of the tick salivary protein evasin-4

**DOI:** 10.1074/jbc.RA120.013891

**Published:** 2020-08-14

**Authors:** Stepan S. Denisov, Mercedes Ramírez-Escudero, Alexandra C. A. Heinzmann, Johannes H. Ippel, Philip E. Dawson, Rory R. Koenen, Tilman M. Hackeng, Bert J. C. Janssen, Ingrid Dijkgraaf

**Affiliations:** 1 Department of Biochemistry, Cardiovascular Research Institute Maastricht, Maastricht University, Maastricht, The Netherlands; 2 Department of Crystal and Structural Chemistry, Bijvoet Center for Biomolecular Research, Faculty of Science, Utrecht University, Utrecht, The Netherlands; 3 Department of Chemistry, The Scripps Research Institute, La Jolla, California, USA

**Keywords:** chemokine, nuclear magnetic resonance (NMR), protein structure, protein–protein interaction, X-ray crystallography, parasite, immunosuppressor, ticks

## Abstract

Ticks, as blood-sucking parasites, have developed a complex strategy to evade and suppress host immune responses during feeding. The crucial part of this strategy is expression of a broad family of salivary proteins, called Evasins, to neutralize chemokines responsible for cell trafficking and recruitment. However, structural information about Evasins is still scarce, and little is known about the structural determinants of their binding mechanism to chemokines. Here, we studied the structurally uncharacterized Evasin-4, which neutralizes a broad range of CC-motif chemokines, including the chemokine CC-motif ligand 5 (CCL5) involved in atherogenesis. Crystal structures of Evasin-4 and E66S CCL5, an obligatory dimeric variant of CCL5, were determined to a resolution of 1.3–1.8 Å. The Evasin-4 crystal structure revealed an L-shaped architecture formed by an N- and C-terminal subdomain consisting of eight β-strands and an α-helix that adopts a substantially different position compared with closely related Evasin-1. Further investigation into E66S CCL5–Evasin-4 complex formation with NMR spectroscopy showed that residues of the N terminus are involved in binding to CCL5. The peptide derived from the N-terminal region of Evasin-4 possessed nanomolar affinity to CCL5 and inhibited CCL5 activity in monocyte migration assays. This suggests that Evasin-4 derivatives could be used as a starting point for the development of anti-inflammatory drugs.

Ticks are a group of obligatory blood ectoparasites that are found all over the world counting ∼900 species representing three families: soft ticks *Argasidae* (∼200 species), hard ticks *Ixodidae* (∼700 species), and monotypic *Nuttalliellidae* (1, 2). Although ticks are generally considered to be pests that transmit tick-borne diseases and cause economic losses for animal husbandry, they can be used as a valuable source of new molecules for drug development. Indeed, because of their parasitic nature, ticks have developed numerous bioactive compounds in the course of evolution to avoid immune responses and battle hemostasis while obtaining a blood meal (3). To achieve this, ticks target almost every key player in the host innate and adaptive immune system such as neutrophils, B and T cells, and dendritic cells (4). Trafficking and recruitment of monocytes, neutrophils, T lymphocytes, and other responsive cells are orchestrated by a family of chemotactic cytokines, termed chemokines. Chemokines constitute the largest family of cytokines and are divided into four subgroups (C, CC, CXC, or CX3C), according to the number and arrangement of conserved cysteines. All but three of the human chemokines fall either in the CC or CXC group and consist of 70–80 amino acids (5).

Disruption of the chemokine signaling system is one of the crucial parts of ticks' strategy to evade immune responses. To neutralize chemokines, ticks secrete small cysteine-rich chemokine-binding proteins in their saliva with no homologues in mammals, termed Evasins (6). Evasins can be divided into two structurally unrelated C6 or C8 subfamilies embodying six and eight cysteines, respectively (6). C8-Evasins are high-affinity binders of CC-type chemokines, whereas C6-Evasins bind CXC-type chemokines (7). Although several hundred of putative C6- and C8-Evasins have been identified in tick sialomes (8–12), only a few of them have been studied in detail, especially from a structural point of view. The first identified and structurally characterized family member is Evasin-1, which was isolated from the brown dog tick, *Rhipicephalus sanguineus* (10). Evasin-1 is a 94–amino acid protein from the C8 subfamily and a high-affinity binder of closely related CCL3, CCL4, and CCL18 CC-motif chemokines. Structures of this protein and its complex with CCL3 have been resolved by X-ray crystallography (13).

In contrast to the highly selective Evasin-1, its homologue Evasin-4 binds ∼20 CC-chemokines with nanomolar affinity (11). Among this broad range of chemokines, the chemokine CC-motif ligand 5 (CCL5, also known as RANTES) is of particular interest, because it is one of the most expressed chemokines by activated platelets and plays a prominent role in the development of atherosclerosis (14). CCL5 activates receptors CCR5, CCR3, and CCR1 and mediates monocyte arrest on inflamed endothelium (15). CCL5 activity is regulated by formation of CCL5 oligomers (16) and heterodimers with other chemokines (17, 18). For example, heterodimerization of CCL5 with CXCL4 (platelet factor 4) has a synergetic effect on monocyte adhesion, whereas disruption of this heterodimer attenuates atherosclerosis development (19, 20).

It has been shown that treatment with Evasin-4 reduces cardiac injury and inflammation in mice because of anti-CCL5 activity (19). However, the structure of Evasin-4 and its binding mode to chemokines remain unresolved. Here, we present the crystal structure of Evasin-4 at 1.3 Å resolution, which presents new structural features within the Evasin family members. We also identified the structural determinants of the CCL5–Evasin-4 complex by solution NMR spectroscopy and the inhibitory role of Evasin-4 and its N terminus on CCL5-induced monocyte migration.

## Results

### Expression and purification of Evasin-4

Evasins are reported to be highly glycosylated when expressed in insects or mammalian cells; however, glycosylation is not crucial for their activity (8, 13). To obtain a more homogeneous sample for NMR and X-ray studies, Evasin-4 was expressed in a nonglycosylated form in *Escherichia coli*. The molecular mass of the expressed polypeptide was 11.4 kDa, indicating the retention of the N-terminal methionine in Evasin-4 (designated as met-Evasin-4). Oxidative refolding of this protein led to a decrease in molecular mass of 8 Da, corresponding to the formation of four disulfide bonds. Purification by HPLC yielded 1–1.5 mg of met-Evasin-4 per liter of culture (Fig. S1). In an unsuccessful attempt to increase expression yield, Evasin-4 was expressed as a fusion protein with an N-terminal His_6_-SUMO tag, resulting in ∼1 mg/liter of Evasin-4 after cleavage by SUMO protease and subsequent refolding and purification by HPLC (Fig. S2). Both Evasin-4 and met-Evasin-4 were used for further experiments as specified under “Experimental procedures.” To avoid confusion, both variants are called Evasin-4 throughout the manuscript because no structural and functional differences between them are expected.

**Figure 1.**
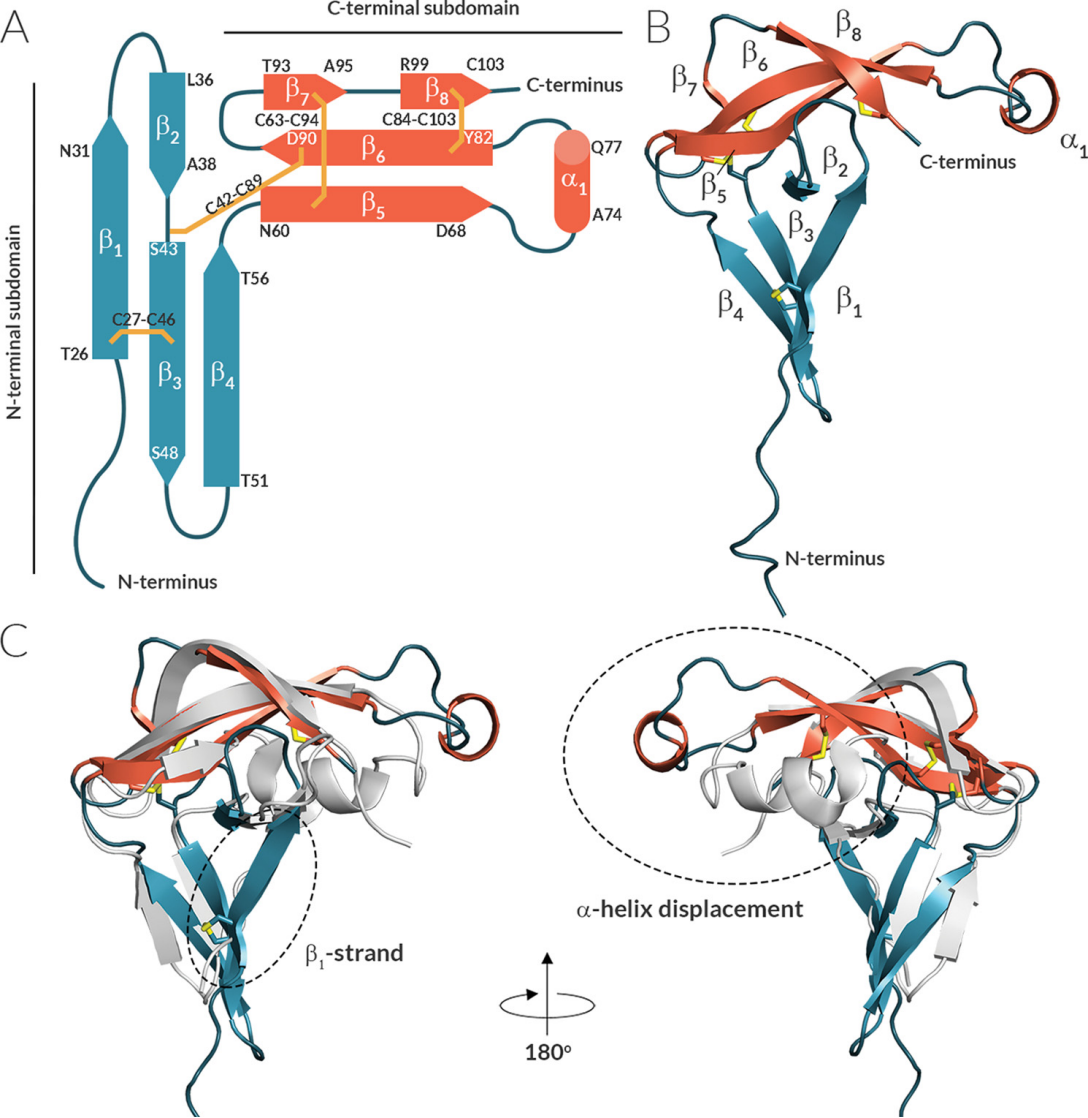
**Evasin-4 contains a core formed by two orthogonal β-sheets.** *A* and *B*, schematic representation of the secondary structural elements of Evasin-4 (*A*) and cartoon representation of its crystal structure (*B*). β-Strands are depicted as *arrows*, α-helixes are *cylinders*, disulfide bonds are *yellow lines*. *C*, superposition of Evasin-1 (PDB code 3FPR, *gray*) and Evasin-4 (PDB code 6ST4, colored according to structures in *A*). The different position of the α-helix in Evasin-1 and Evasin-4 is highlighted by a *dashed circle*.

**Figure 2.**
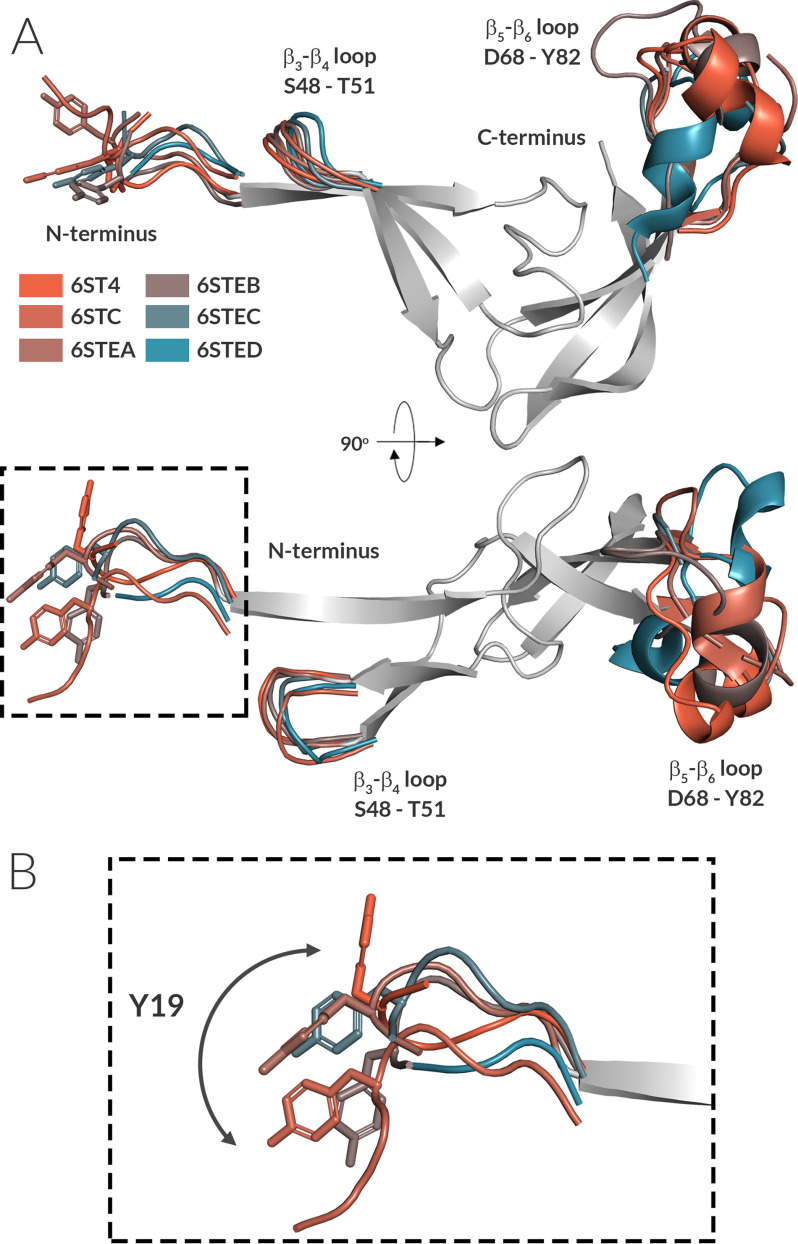
**The rigid Evasin-4 core is flanked by three more flexible regions.** *A*, front and top view of the superposition of six independent Evasin-4 monomers derived from three crystal forms. For clarity, only a single core structure is shown (*gray*), with flexible regions displayed and differently colored for each monomer. α-Carbon superposition gives a RMSD of 1.2 Å between the six monomers of the three crystal structures. *B*, enlarged image (*square* in *A*) showing the conformational plasticity of the Evasin-4 N terminus. Tyr^19^, known to be decisive for binding to CCL5, is highlighted in *stick representation*.

### Structure determination of Evasin-4

To study Evasin-4's structure by NMR spectroscopy, metabolically ^15^N- and ^13^C-enriched Evasin-4 was produced. However, Evasin-4 appeared to be a challenging protein for structure determination by solution NMR spectroscopy because a significant portion of the protein resonances were extremely broadened at 37 °C and pH 7.1. Unfortunately, varying the conditions such as ionic strength, pH, and temperature did not improve the quality of the spectra. In the ^15^N-^1^H HSQC spectrum of 50 μm [^15^N,^13^C] Evasin-4, only ∼40% of the backbone amide peaks could be observed and assigned, namely the Asp^12^–Ala^23^, Phe^69^–Gln^80^ and His^88^–Thr^93^ regions, and several other residues scattered along the sequence (see Fig. S3).

**Figure 3.**
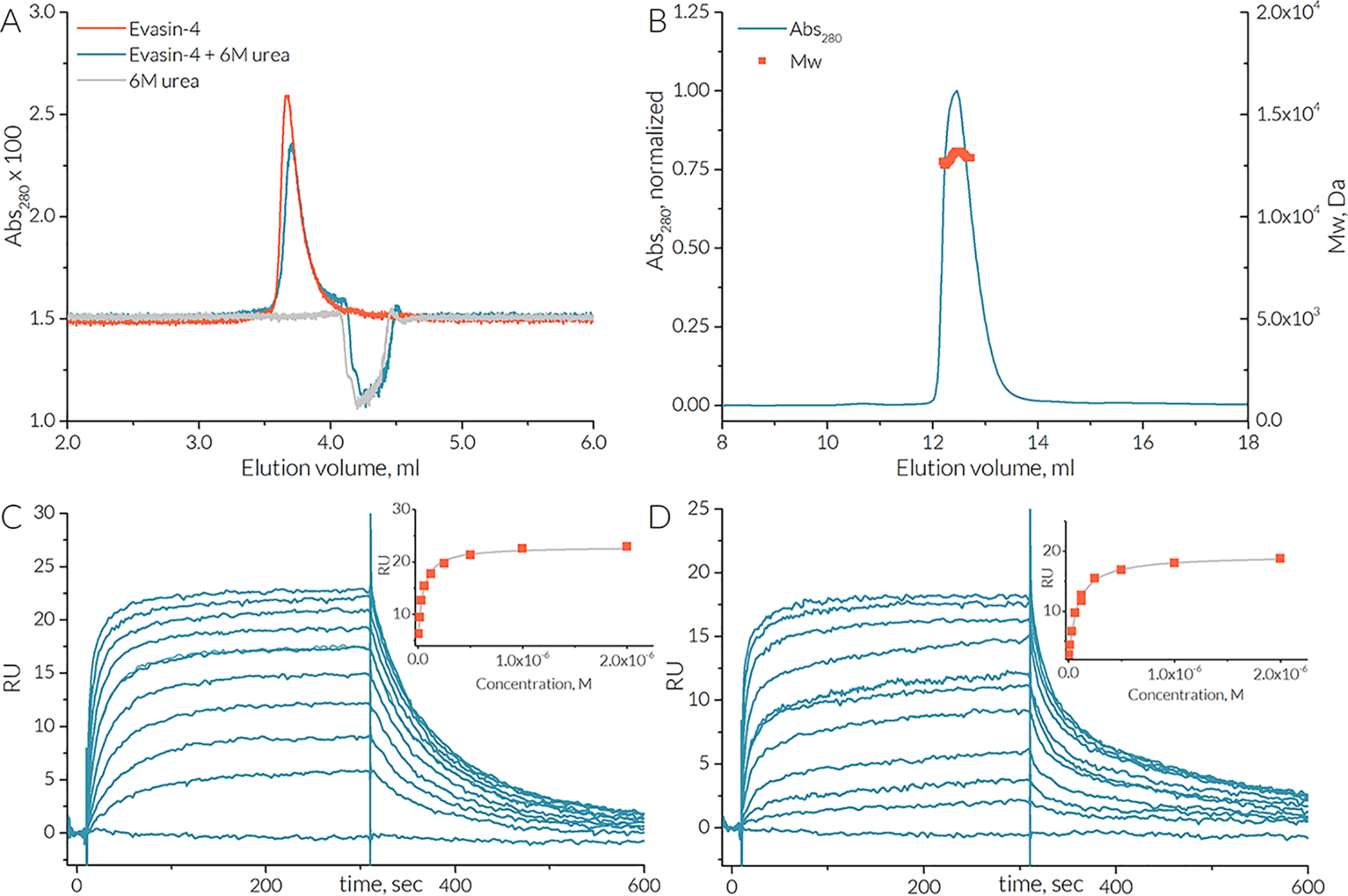
**Evasin-4 is a monomer in solution and stoichiometrically binds one monomer of CCL5.** *A*, SEC traces of 0.1 mg/ml Evasin-4 in the presence (*blue line*) and absence (*orange line*) of 6 m urea. *B*, SEC-MALS analysis of Evasin-4. Evasin-4 elutes at a concentration of 1 mg/ml as a single peak (*blue line*) with the average molecular mass of 12.9 ± 1.2 kDa (*orange squares*). *C* and *D*, SPR biosensor analysis: Evasin-4 binding to E66S CCL5 (*C*) and [NMe-^7^T]CCL5 (*D*). The binding curves are plotted using maximal response signal for each injection. The *K_D_* values are calculated by fitting data to the steady-state affinity model and are found to be 42 and 75 nm for E66S CCL5 and [NMe-^7^T]CCL5, respectively.

To bypass the challenges confronted during NMR experiments, we determined the structure of Evasin-4 by X-ray diffraction. Unlabeled Evasin-4 crystallized in three different crystal forms (Protein Data Bank (PDB) codes 6ST4, 6STC, and 6STE) with the best crystals diffracting to a maximum resolution of 1.3 Å (Table S2). Phasing of the diffraction data by molecular replacement using the closest homologous structure available, Evasin-1 (13) (PDB code 3FPR, 27% sequence identity), as a full template (20, 21) or as fragments (22), was unsuccessful. In the end, to phase the data, a single-wavelength anomalous dispersion data set was collected at a wavelength of 1.54 Å. This strategy provided sufficient anomalous signal arising from the eight sulfur atoms of cysteine residues that enabled phasing of the Evasin-4 data.

The overall architecture of the Evasin-4 can be divided into an N- and C-terminal subdomain (Fig. 1). Each of the N- and C-terminal subdomains consists of a three-stranded anti-parallel β-sheet core, in which the sheets are oriented orthogonally with respect to each other, forming an L-shaped structure. The N-terminal β-sheet is formed by β-strands β–β4, whereas the C-terminal β-sheet constitutes β-strands β5–β8. The C-terminal subdomain has a triangle-like twisted-sheet shape with a short α-helix (Ala^74^–Gln^77^) within the β5–β6 loop that is positioned away from the rest of the structure (Fig. 1*C*). The 3D fold of Evasin-4 is tightly arranged by four disulfide bonds and contains a rigid protein core. Three disulfide bonds connect β-strands within a subdomain (Cys^27^–Cys^46^ connects β1 and β3, Cys^63^–Cys^94^ links β5 and β7, and Cys^84^–Cys^103^ connects β6 and β8), whereas the fourth disulfide bond (Cys^42^–Cys^89^) joins the N-terminal subdomain with the β6-strand of the C-terminal subdomain. Superposition of all six polypeptide chains of the three Evasin-4 crystal forms showed that the rigid Evasin-4 protein core is flanked by two more flexible protrusions (Fig. 2). The first protrusion is formed by the N-terminal region (Glu^16^–Leu^25^) plus the loop β3–β4 (Ser^48^–Thr^51^) that extend from the N-terminal β-sheet. The second protrusion extends from the C-terminal β-sheet and consist of the loop β5–β6 (Asp^68^–Tyr^82^), including the α-helix (Ala^74^–Gln^77^). Residues Glu^1^–Glu^15^ are unresolved in all three crystal forms as a result of high flexibility. In addition, loop β5–β6 is unresolved in two of the total six monomers in the three crystals forms (residues Gln^72^–Asn^79^ in chain A and Thr^71–^Gln^80^ in chain C in crystal form 3; PDB code 6STE).

The crystal packing reveals a large intermolecular interface of 2,380 Å^2^ buried surface area common to all three Evasin-4 crystal forms. This interface involves the N-terminal region of Evasin-4 that forms an extended intermolecular antiparallel β-sheet, predominantly involving strand β1 next to strands β3 and β4 of each molecule (Fig. S4). However, size-exclusion chromatography (SEC) and multiangle light scattering (MALS) analysis in solution (Fig. 3*C*) show that Evasin-4 is a monomer, indicating that the packing in the crystal may not be physiologically relevant. First, Evasin-4 was analyzed by SEC at a concentration of 0.1 and 1 mg/ml, showing a single peak (Fig. 3*A*). Addition of 6 m urea did not affect the Evasin-4 peak position, indicating that Evasin-4 remained in the lowest oligomeric state. To identify the molecular mass of Evasin-4 in solution, SEC-MALS analysis was performed. At neutral pH and a concentration of 5.3 mg/ml at injection (which dilutes to 1 mg/ml at elution), Evasin-4 showed a single peak with a molecular mass of 12.9 ± 1.2 kDa (Fig. 3*B*), which corresponds to a monomeric state (mass of a monomer is 11.3 kDa; Fig. S2). Although the concentration of Evasin-4 when injected into the host blood stream has not been determined yet, the total protein concentration in saliva of another tick, *Rhipicephalus microplus*, has been reported to range from 1.75 to 3.22 mg/ml (23). Thus, one can conclude that Evasin-4 is a functional monomer, whereas the observed dimer interactions in the crystal structure are a result of crystal packing.

**Figure 4.**
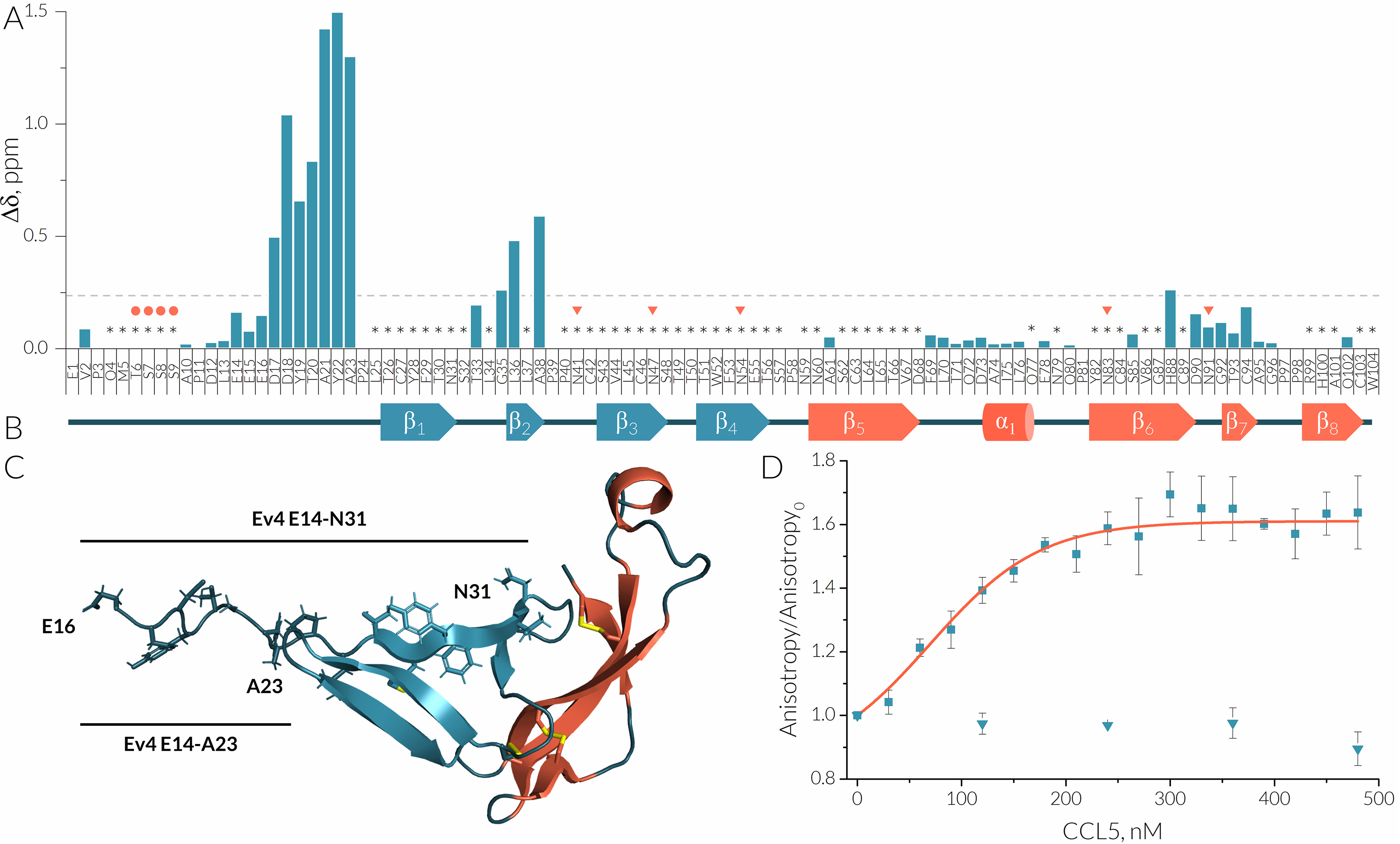
**Binding of CCL5 is mediated via the N terminus of Evasin-4.** *A*, the CSP plot of 100 μm E66S CCL5/[^15^N,^13^C, ^2^D]Evasin-4 complex and a reference sample 50 μm [^15^N,^13^C]Evasin-4 at 37 °C, pH 7.1. Δ values are expressed as the sum of square roots Δ chemical shifts of ^1^H and ^15^N (weighted according to [0.25*Δ^15^N]^2^ and [Δ^1^H]^2^ in ppm). Missing signals are marked by *asterisks*, and predicted (47, 48) *O*- and *N*-glycosylation sites are marked by *circles* and *triangles*, respectively. *B*, a schematic representation of the secondary structure of met–Evasin-4 extracted from the crystal structure 6ST4. *C*, peptides derived from the N-terminal region of Evasin-4 marked on the crystal structure 6ST4. *D*, titration of 100 nm 5(6)-Fam–labeled Ev4 Glu^14^–Asn^31^ (*rectangles*) and Ev4 Glu^14^–Ala^23^ (*circles*) by CCL5 followed by fluorescence anisotropy. The apparent *K_D_* value for Ev4 Glu^14^–Asn^31^ calculated from the fitted titration curve (*orange*) is 69 ± 12 nm.

We cannot exclude the possibility that the crystal packing has an effect on the conformation of Evasin-4. However, the overall topology is conserved comparing Evasin-4 with Evasin-1 (Fig. 1*C*). In addition, the six independent structures of Evasin-4, arising from three different crystal forms we report here, are very similar to each other (Fig. 2), suggesting that the crystal packing does not have major consequences on the overall structure of Evasin-4.

### Evasin-4 binding to CCL5

CCL5 can oligomerize, forming dimers and oligomers of a higher order (16), which would complicate analysis of Evasin-4 binding. To prevent higher-order CCL5-oligomerization, the E66S CCL5 mutant was used for further experiments, as it mainly forms dimers at physiological pH (24). Although the similar E66A CCL5 mutant also showed deficiency in oligomerization without disturbing Evasin-4 inhibition potency (25), it is still not clear whether Evasin-4 binds monomer or dimer CCL5. To address this question, surface plasmon resonance (SPR) biosensor analysis was performed with biotinylated Evasin-4 as a ligand immobilized on the chip surface. Dimeric E66S CCL5 and a synthetic obligatory CCL5 monomer (Figs. S5 and S6) that has a methylated amide nitrogen at Thr^7^ ([NMe-^7^T]CCL5) (26) were used as analytes. E66S CCL5 bound Evasin-4 in a dose-dependent manner with an apparent *K_D_* value of ∼42 nm (Fig. 3, *C* and *D*). The monomeric [NMe-^7^T]CCL5 variant was effectively bound by Evasin-4 as well, with an apparent *K_D_* value of ∼75 nm. These similar *K_D_* values could indicate that the CCL5 monomer is the main Evasin-4 target.

**Figure 5.**
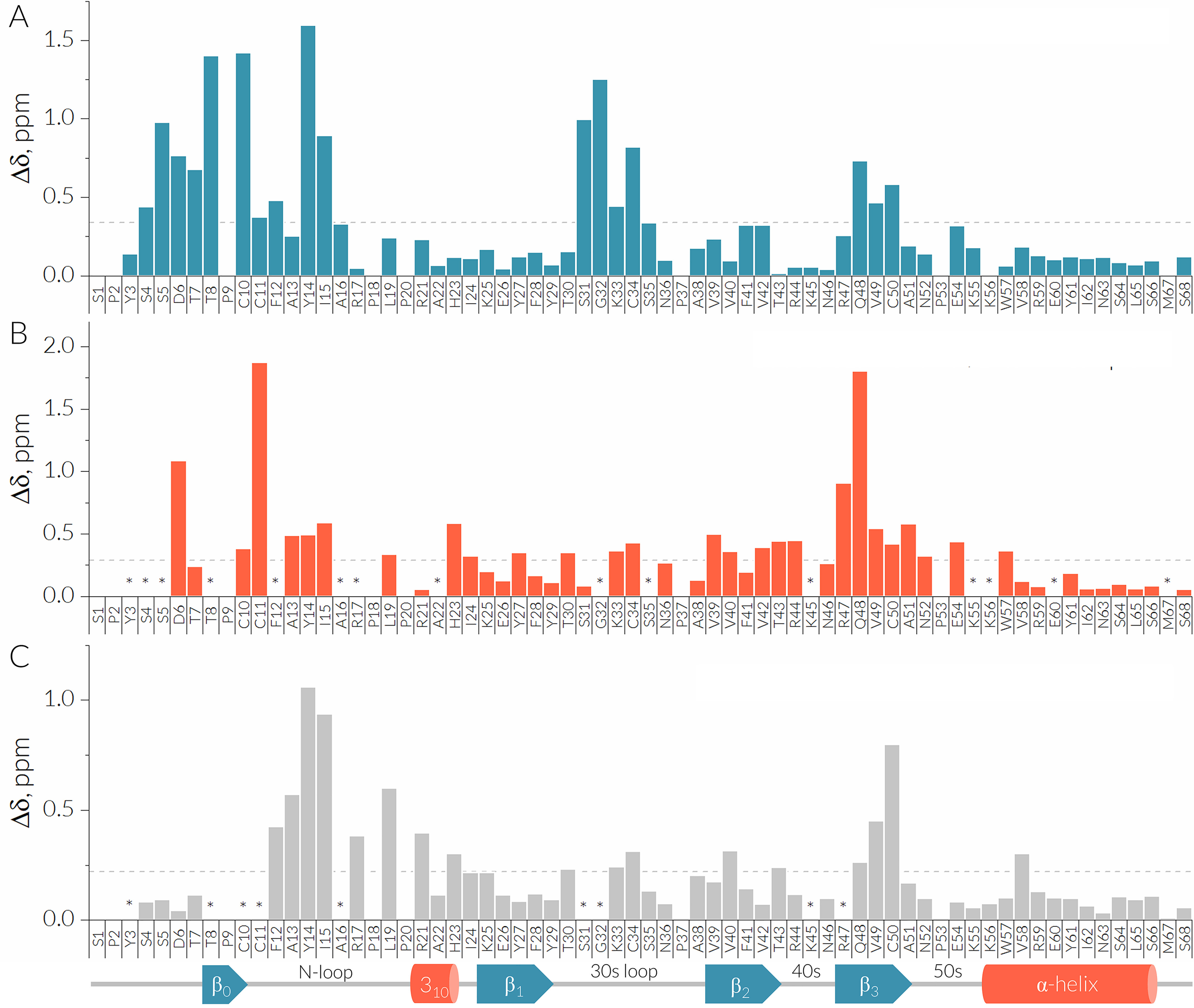
**Binding of Evasin-4 is mediated through the CCL5 dimer interface.** The CSP plots between the monomeric and dimeric form of [^15^N, ^13^C] E66S CCL5 at a concentration of 20 μM at 37°C, pH 4.5 (A); the 20 μM dimer form of [^15^N, ^13^C] E66S CCL5 and 80 μM [^15^N, ^13^C] E66S CCL5/Evasin-4 complex at 37°C, pH 7.0 (B); 25 μM monomer form of [^15^N] E66S CCL5 before and after addition of 500 μM Ev4 Glu^14^-Asn^31^ at 37°C, pH 4.5 (C). The Δ values are expressed as the sum of square roots delta chemical shifts of ^1^H and ^15^N (weighted according to [0.25*Δ^15^N]^2^ and [Δ^1^H]^2^ in ppm). Missing signals are marked by *asterisks*. A schematic representation of the secondary structure of E66S CCL5 extracted from the crystal structure is shown at the *bottom* of the panel.

**Figure 6.**
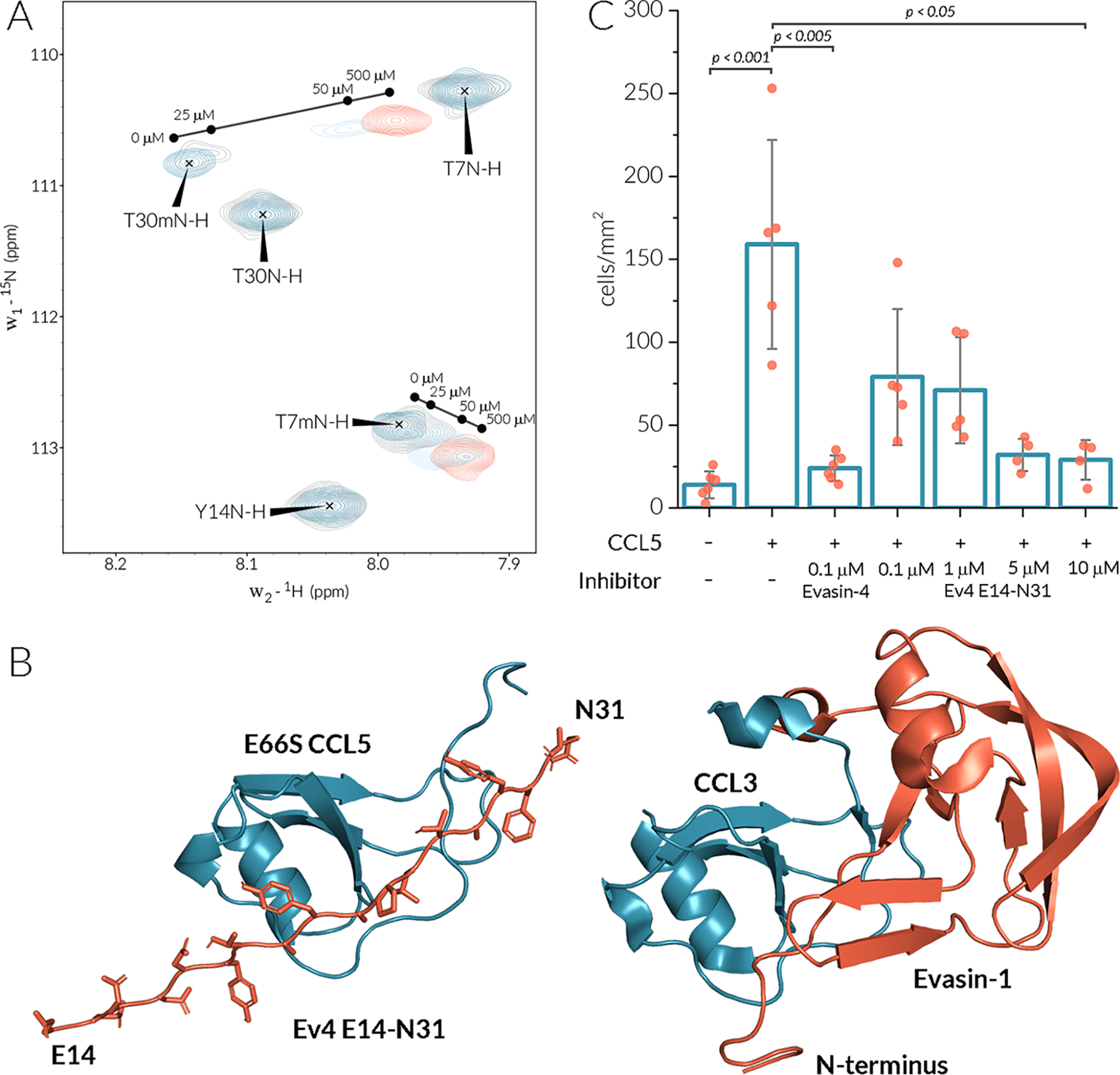
**Ev4 Glu^14^–Asn^31^ binds the CCL5 monomer and inhibits monocyte migration.** *A*, the section of ^15^N-^1^H HSQC spectra of 25 μm ^15^N E66S CCL5 at different concentrations of Ev4 Glu^14^–Asn^31^. Signals of the monomeric form of E66S CCL5 are designated by a *letter m*. *B*, the HADDOCK model of the E66S CCL5/Ev4 Glu^14^–Asn^31^ complex (*left panel*) and the crystal structure 3FPU of the CCL3–Evasin-1 complex (*right panel*). *C*, inhibition of THP-1 cells (1 × 10^6^ cells/ml) migration toward CCL5 (0.5 µg/ml) by Evasin-4 and increasing concentrations of Ev4 Glu^14^–Asn^31^.

For in-depth analysis of the Evasin-4/CCL5 complex, determination of its crystal structure was attempted by X-ray crystallography. Unfortunately, several trials to crystallize Evasin-4 in complex with E66S CCL5 were unsuccessful, primarily because of the precipitation of the complex in solution, even at a low concentration. This could be explained by the large difference in p*K_i_* values of Evasin-4 and E66S CCL5 and their limited solubility at different pH. Whereas Evasin-4 is an acidic protein with a theoretical p*K_i_* value of 3.8, E66S CCL5 has a basic p*K_i_* value of 9.4. This greatly limited the choice of conditions at which both Evasin-4 and E66S CCL5 remained in solution. Although solubility was improved to some extent by increasing the amount of salt in the protein buffer or adding organic solvents such as glycerol, crystals that were obtained in these trials consisted of E66S CCL5 only. These crystals were used to solve the E66S CCL5 structure by X-ray crystallography. As expected, E66S CCL5 formed a dimer and had a structure very similar to that of WT CCL5 with a root-mean-square deviation (RMSD) of 0.63 Å for the backbone of the full-length protein (Fig. S7). This confirmed that the E66S mutation disturbed neither the CCL5 folding nor the formation of a dimer.

Because crystals of the E66S CCL5–Evasin-4 complex could not be obtained, NMR spectroscopy was used to further study the complex formation. Addition of E66S CCL5 to [^15^N,^13^C]Evasin-4 caused substantial chemical shift perturbations (CSPs) for the ^15^N-^1^H HSQC spectrum peaks. NMR spectra of the final E66S CCL5–[^15^N,^13^C]Evasin-4 complex showed numerous new resonance signals of Evasin-4 in the complex (Fig. S3*B*). However, a large number of Evasin-4 resonances in the ^15^N-^1^H HSQC spectrum still remain significantly broadened in the complex and could not be assigned by 3D triple resonance spectra or NOESY spectra. Assignment of the observed signals showed that the most perturbed residues are located in the Asp^17^–Ala^23^ and Thr^33^–Ala^38^ regions, whereas residues of the α-helix and strands β6 and β7 remained unaffected (Fig. 4, *A* and *B*).

Because most perturbed residues are located in the N-terminal region of Evasin-4, this region could be responsible for chemokine binding. To assess this hypothesis, two peptides taken from the Evasin-4 sequence were synthesized by Boc-based solid-phase peptide synthesis (Figs. S8 and S9). The first peptide, Ev4 Glu^14^–Ala^23^, comprises the Glu^14^–Ala^23^ residues of Evasin-4, whereas the second one, Ev4 Glu^14^–Asn^31^, includes the longer region Glu^14^–Asn^31^ (Fig. 4*C*). Cys^27^ in the Ev4 Glu^14^–Asn^31^ was mutated to alanine to prevent oxidation and formation of intermolecular disulfide bonds. Affinity of obtained peptides to CCL5 was studied by fluorescence anisotropy using 100 nm of 5(6)-Fam–labeled peptides and human CCL5. Titration of Ev4 Glu^14^–Asn^31^ by human CCL5 caused dose-dependent increase of fluorescence anisotropy, whereas addition of CCL5 to the shorter variant Ev4 Glu^14^–Ala^23^ had no effect even at concentration of 500 nm (Fig. 4*D*). The apparent *K_D_* value of Ev4 Glu^14^–Asn^31^ was calculated to be 69 ± 12 nm.

To study the structural determinants of E66S CCL5 involved in Evasin-4 binding, NMR spectra were recorded for the [^15^N,^13^C]E66S CCL5–Evasin-4 complex. Initial ^15^N-^1^H HSQC spectra of [^15^N,^13^C]E66S CCL5 were recorded at pH 4.5 and 37 °C at concentrations of 1 and 20 μm. At 1 μm, E66S CCL5 is present in a monomeric form, whereas at 20 μm, two sets of signals were observed, corresponding to a monomeric and dimeric form (Fig. S10). The chemical perturbation plot of monomer/dimer equilibria obtained from the ^15^N-^1^H HSQC spectrum of 20 μm E66S CCL5 at pH 4.5, showed that dimerization mostly affected residues of the N-terminal region (Ser^4^–Ile^15^), 30s loop (Ser^31^–Cys^34^), and β3-strand (Arg^47^–Cys^50^) (Fig. 5, *A* and *C*). This observation goes in line with the obtained crystal structure because the E66S CCL5 dimer interface is formed by interactions of the N terminus with the 30s loop and β3-strand.

Subsequently, [^15^N,^13^C]E66S CCL5 was titrated with unlabeled Evasin-4 at pH 4.5. Similar to the crystallization experiments, the titration resulted in protein precipitation and loss of signal that prevented recording of high-quality NMR spectra. Final NMR spectra of [^15^N,^13^C]E66S CCL5 and its complex with Evasin-4 were recorded at pH 7.0–7.3, at which both proteins and the complex are in a soluble form (Fig. S11). However, departing from the optimal pH value of 4.5 for recording of NMR spectra of E66S CCL5 led to signal broadening and loss of several amide signals. Although a high concentration of [^15^N,^13^C]E66S CCL5 (82 μm) was used to minimize these adverse effects, 16 amide peaks were missing in the ^15^N-^1^H HSQC spectrum of [^15^N,^13^C]E66S CCL5 and [^15^N,^13^C]E66S CCL5–Evasin-4 complex at pH 7.0–7.3. The addition of a slight excess of met–Evasin-4 resulted in a single set of signals in the ^15^N-^1^H HSQC spectra of [^15^N,^13^C]E66S CCL5, indicating 1:1 stoichiometry of the complex. (Fig. S7*B*). The chemical shift perturbation plot between the [^15^N,^13^C]E66S CCL5 dimer and the [^15^N,^13^C]E66S CCL5–Evasin-4 complex showed that the most perturbed residues by Evasin-4 binding are located in the N-terminal region (Asp^6^, Cys^11^, Ala^13^–Ile^15^), β_2_-strand (Val^39^–Thr^43^), and β_3_-strand (Arg^47^–Ala^51^) (Fig. 5*B*). The affected residues comprised a continuous surface in the 3D structure of E66S CCL5, coinciding with the E66S CCL5 dimer interface, which suggests that binding of Evasin-4 causes disruption of the CCL5 dimer.

Analogous to the complex of E66S CCL5 with full-length Evasin-4, NMR spectra were recorded for the complex of CCL5 with the Ev4 Glu^14^–Asn^31^ peptide. Although Ev4 Glu^14^–Asn^31^ is poorly soluble at low pH values because of the presence of negatively charged residues, its complex with E66S CCL5 appeared to be more soluble compared with the E66S CCL5–Evasin-4 complex. This allowed recording of NMR spectra at near optimal conditions for E66S CCL5 when using a high concentration of Ev4 Glu^14^–Asn^31^ to circumvent precipitation of the peptide. Titration of 25 μm [^15^N]E66S CCL5 by increasing concentrations of Ev4 Glu^14^–Asn^31^ caused a gradual shift of signals of monomeric E66S CCL5 and decrease of signal intensity for the dimeric form (Fig. 6*A*). At pH 4.5 the resulting set of signals could be compared with chemical shifts of the monomeric form to reveal the E66S CCL5–Evasin-4 binding interface. The obtained CSP plot (Fig. 5*C*) indicates that most perturbed residues are located in the N-loop (Phe^12^–Arg^21^ and His^23^) and in the β_3_-strand (Gln^48^ and Cys^50^).

The CSP data of E66S CCL5 binding by Ev4 Glu^14^–Asn^31^ was used to model a chemokine/peptide complex using the HADDOCK2.2 web server (27). The crystal structure of E66S CCL5 and a generated linear peptide, Ev4 Glu^14^–Asn^31^, were used as a starting model. For docking, the Phe^13^–His^23^ and Gln^48^–Cys^50^ regions of E66S CCL5 and the Asp^17^–Asn^31^ region of the peptide were set as active residues. The obtained model (Table S3) of the E66S CCL5/Ev4 Glu^14^–Asn^31^ complex (Fig. 6*B*) indicated that the peptide binds in the groove between the N-loop and β3-strand of CCL5 and that the C terminus of the peptide interacts with the N-terminal region of CCL5.

To translate these results into a biologically more relevant system, cell migration of human THP-1 monocytic cells was investigated. CCL5 induced significant migration of THP-1 cells compared with the control in the absence of chemoattractants (Fig. 6*C*). The addition of 100 nm of Evasin-4 effectively blocked chemotaxis of THP-1 cells. Although Ev4 Glu^14^–Asn^31^ showed dose-dependent inhibition of CCL5-induced THP-1 cell migration, the required concentrations were higher compared with the full-length protein, and full inhibition was reached only at a concentration of 10 μm.

## Discussion

Ticks employ a complex mixture of secreted proteins and bioactive compounds to evade host defense mechanisms and to remain unnoticed by the host (3, 4). To neutralize chemokines, ticks produce small chemokine-binding proteins, known as Evasins (28). Although the number of identified Evasins are growing, structural data about them are still scarce and restricted to the structures of Evasin-1 and Evasin-3 from the structurally unrelated C8 and C6 families, respectively (12, 13, 29). In the present study, the structure of Evasin-4 was determined by X-ray crystallography.

The Evasin-4 structure consists of eight β-strands and one short α-helix and has an L-shaped topology with the N- and C-terminal subdomains oriented orthogonally (13). Despite the conserved disulfide linkages and the L-shaped architecture, Evasin-1 and Evasin-4 structures showed a RMSD for Cα atoms of 2.8 Å for the full-length structures. The Evasin-4 structure is bulkier (approximately 30 × 50 × 50 Å compared with 13 × 20 × 35 Å for Evasin-1 (13)), mainly because of the alignment of the β5–β6 loop and the N-terminal subdomain of Evasin-4 in the same plane (Fig. 1*C*). This difference explains why a direct molecular replacement using the Evasin-1 structure as a template was unsuccessful to solve the Evasin-4 crystal structure.

Evasin-1 has an extension at the C terminus formed by a short α-helix (α_2_) that is involved in chemokine binding. The α-helix in Evasin-4 is displaced and does not interact with the N-terminal subdomain, as observed in Evasin-1. It has been proposed that interactions of the residues of this α-helix (Trp^89^ and Arg^90^) with the N-terminal residues of CCL3 could serve as an “address” that drives the narrow selectivity of Evasin-1 (13). The C terminus of Evasin-4 has no flexible region and is rigidly embedded in the protein core by the Cys^84^–Cys^103^ disulfide bond. This points to an inability of Evasin-4 to accommodate chemokines through interaction with the C-terminal subdomain, in contrast to Evasin-1. This consideration goes in line with alanine scanning data, which revealed that the C-terminal Trp^103^ and residues Gln^72^ and Gln^77^ in the α-helix region of Evasin-4 appear to be nonessential for CCL5 binding (25). Taking into account that no perturbations of chemical shifts in the C-terminal subdomain are observed upon E66S CCL5 binding, one could then hypothesize that the N-terminal subdomain of Evasin-4 is the driving force of selectivity and chemokine binding.

The N-terminal regions of Evasin-4 and Evasin-1 were shown to form most of the contacts with chemokines (13, 25). The importance of the N terminus for chemokine binding is supported by the E66S CCL5-induced pattern of chemical shift perturbations of Evasin-4, which indicated that most of the affected residues are located in the N-terminal region Asp^17^–Ala^23^. In fact, the N terminus of C8-Evasins seems to play a crucial role in chemokine binding. For instance, replacement of the N terminus of Evasin-1 by that of P672, another C8-Evasin with different chemokine selectivity, produced a chimera with a gain in affinity to CCL8, which is normally not a typical binding partner of native Evasin-1 (30). Furthermore, the linear peptide Ev4 Glu^14^–Asn^31^, derived from the Evasin-4 sequence, possesses nanomolar affinity to CCL5 on its own. Similar tight affinities have recently been shown for peptides derived from the N terminus of P672 and are potent binders of CCL8 (31). The N-terminal region of Evasin-4 embodies multiple tyrosines, which are proposed to mimic an N-terminal region of CC-chemokine receptors (32). Indeed, the Y19A mutation was shown to dramatically decreased the binding of Evasin-4 to CCL3, CCL5, and CCL8 (25). Moreover, Tyr^19^ and Tyr^21^ are predicted to undergo post-translational sulfation that could further improve affinity to chemokines (32). However, the short peptide Ev4 Glu^14^–Ala^23^, which also contains both tyrosines, showed no affinity to CCL5, which indicates that residues from Pro^24^ to Asn^31^ contribute to chemokines binding as well.

The physiological activity of CCL5 is tightly regulated by its oligomeric state (16). It has been demonstrated that CCL5 oligomerization is required for CCR1 but not for CCR5 activation (17). The CCL5 dimer is formed through intermolecular interactions of the CCL5 N termini comprising a short anti-parallel β-strand, whereas higher order oligomeric states are assembled by dimer–dimer contacts of the 40s loop with the α-helix and β_1_-strand (16, 33). The Evasin-4–induced chemical shift perturbation pattern of E66S CCL5 coincides with the CCL5 dimer interface, which is an indication of dimer disruption and, therefore, a 1:1 CCL5–Evasin-4 complex formation. This hypothesis is supported by the ability of Evasin-4 to bind the obligatory monomeric [NMe-^7^T]CCL5. Moreover, chemical shift perturbations induced by the Ev4 Glu^14^–Asn^31^ showed that only CCL5 monomer signals are affected upon binding. Thus, Evasin-4 binds to the CCL5 monomer and causes disruption of the CCL5 dimer by shifting the monomer/dimer equilibrium toward the monomeric form of the chemokine and not by direct replacing of CCL5 from the dimer. A similar mode of chemokine binding was recently shown for Evasin-3 (29). The model of the E66S CCL5/Ev4 Glu^14^–Asn^31^ complex shows that the position of the peptide is equivalent to that of the N terminus of Evasin-1 in the CCL3–Evasin-1 complex (13). Interestingly, Tyr^19^ and Tyr^22^ of the peptide interact with basic residues Arg^17^ and His^23^, respectively. Thus, post-translational sulfatation could indeed facilitate electrostatic interactions between tyrosines and these basic residues and therefore increase affinity (32).

Although neutralization of CCL5 by Evasin-4 has been shown to reduce cardiac injury and inflammation (19), its application as a therapeutic is highly unlikely because of relatively high molecular mass and complex production. Development of chemokine-binding peptides and peptidomimetics based on Evasins may provide more perspectives (34). Recently, several peptides derived from C8-Evasin P672 were shown to effectively block inflammation because of binding to multiple CC-type chemokines (31). Ev4 Glu^14^–Asn^31^ effectively inhibits CCL5-induced monocyte migration. Taking into account that chemokine-binding peptides attenuate atherosclerosis progression through CCL5 neutralization and CCL5/CXCL4 heterodimer disruption (35), the structural data obtained here could be used as a starting point for development of Evasin-4–based chemokine-binding peptides and peptidomimetics for the treatment of atherosclerosis and inflammation-associated cardiovascular diseases.

In summary, the crystal structures of Evasin-4 are reported with a resolution up to 1.3 Å. Evasin-4 adopts an L-shaped 3D fold similarly to Evasin-1. However, the positions of the N- and C-terminal subdomains are different comparing Evasin-4 to Evasin-1, indicating different modes of chemokine binding. NMR spectroscopy shows that the Evasin-4 N terminus plays an essential role in chemokine binding. The peptide Ev4 Glu^14^–Asn^31^, derived from the N-terminal region of Evasin-4, binds CCL5 with nanomolar affinity and blocks CCL5-induced monocyte migration.

## Experimental procedures

### Protein expression and synthesis

Proteins were expressed or synthesized and subsequently folded as described previously (29). The detailed procedures are described in the supporting information.

### NMR spectroscopy

For NMR sample preparation, lyophilized proteins were dissolved in 25 mm phosphate buffer (pH 6.8–7.1) or 25 mm sodium acetate-d^3^ buffer (pH 4.5) containing 0.1 mm EDTA, 0.2 mm sodium azide, 5% (v/v) D_2_O for deuterium lock, at final concentration of 50 μm for m–t-Evasin-4 and 82, 20, and 1 μm for E66S CCL5. The samples were buffer exchanged four or five times by ultracentrifugation using prewashed Amicon Ultra-3000 3-kDa (Millipore, USA) ultracentrifugation filters. Final NMR samples were prepared in 3- or 5-mm NMR tubes, containing traces of DSS for chemical shift calibration. All NMR spectra were recorded using a Bruker Avance III HD 700 MHz spectrometer, equipped with a cryogenically cooled TCI probe at 37 °C. To make the complex, equimolar amounts of unlabeled met–Evasin-4 or E66S CCL5 were titrated to their [^15^N,^13^C]-enriched complement samples. For met–Evasin-4 and the two complexes, the solution pH of the NMR sample was carefully adjusted to pH 7.1 to ensure stable, nonprecipitating solutions. Backbone resonance assignment of met–Evasin-4 and E66S CCL5 was derived from a combination of ^15^N-^1^H HSQC, 3D HNCO, 3D CACB(CO)NH, 3D HNCACB, and 3D HN(CO)CA spectra recorded at 37 °C.

In the case of E66S CCL5/Ev4 Glu^14^–Asn^31^ complex, 25 μm of ^15^N E66S CCL5 in 25 mm acetate buffer, pH 4.5, was titrated by increasing concentrations of Ev4 Glu^14^–Asn^31^. For each concentration ^15^N-^1^H HSQC was recorded. Backbone resonance assignments were derived by following the shift of peak positions.

### Crystallization, X-ray data collection, and structure determination

Lyophilized met–Evasin-4 and E66S CCL5 were reconstituted in 20 mm Tris, 100 mm NaCl (pH 7.3) and concentrated up to 10 mg/ml. High-throughput screening of commercial crystallization conditions (JCSG Core I to IV suites, Qiagen) was carried out on a Gryphon LCP robot (Art Robbins Instruments) mixing equal volumes of protein and mother liquor (200 nl each) under sitting-drop vapor-diffusion conditions at 20 °C. met–Evasin-4 crystallized in three different forms and mother liquors (crystal form 1, PDB code 6ST4: 40% ethylene glycol, 0.1 m phosphate-citrate, pH 4.2, 0.2 m ammonium sulfate; crystal form 2, PDB code 6STC: 40% PEG 400, 0.1 m sodium acetate, pH 4.5; crystal form 3, PDB 6STE: 25% PEG 4000, 15% glycerol, 85 mm acetate, pH 4.6, 170 mm ammonium acetate). E66S CCL5 crystallized in the same condition as met–Evasin-4 crystal from 3. Several attempts to crystallize the E66S CCL5–met–Evasin-4 complex (1:1 ratio) were unsuccessful.

For data collection, E66S CCL5 crystals were cooled to 100 K in liquid nitrogen before being measured at the Diamond Light Source Synchrotron (Didcot, UK) on Beamline I24. E66S CCL5 crystals have the space group *P*2_1_2_1_2_1_ and contain two molecules in the asymmetric unit, corresponding to a physiological dimer (16). Native diffraction data for met–Evasin-4 crystals were collected at the European Synchrotron Radiation Facility (Grenoble, France) on Beamlines ID29 and ID30-A3. Diffraction images were integrated with XDS (36) or DIALS (37) and subsequently scaled and merged by the Pointless, Aimless, and Ctruncate pipeline in the CCP4 package (38). A summary of data collection and reduction statistics is provided in Table S2. The Evasin-4 structure could not be solved by molecular replacement using the structure of the closest related protein available (Evasin-1, PDB code 3FPR (13), 27% sequence identity). Instead, an anomalous data set was collected in-house on a rotating anode X-ray source (Bruker, wavelength, 1.54 Å) from the same crystals that first provided the native data set. The anomalous data set was processed with EVAL (39) to 2.0 Å resolution, and phases were calculated using SHELX c/d/e of the HKL2map package (40). These data set had good anomalous signal up to 2.8 Å and good quality phases were obtained, possibly because of the relatively high number of sulfur atoms (8 cysteines of 87 residues). Phases were transferred to the native data set (1.29 Å resolution) by molecular replacement using the SHELXe derived model in Phaser (20). A new model was built with autobuilding in ARP/wARP (41), followed by several rounds of manual building in COOT (42) and automatic refinement with REFMAC and Phenix (43, 44). Implementation of bulk-solvent correction and TLS (translation/libration/screw) refinement allowed modeling the protein chain from residue Asp^18^ to Trp^104^. This met–Evasin-4 crystal form 1 structure (PDB code 6ST4) was used to solve the other two crystal forms of met–Evasin-4 by molecular replacement with MOLREP (21). The E66S CCL5 mutant structure was determined by molecular replacement using the structure of the WT CCL5 (PDB code 5COY (16)). At the next steps, solvent molecules and water were added to the structure models which, combined with more rounds of geometry-restrained refinement, led to the final statistics reported in Table S2.

The stereochemistry of the models was checked with MolProbity (45). Coordinates and associated structure factors are deposited in the PDB under accession codes 6ST4, 6STC, 6STE, and 6STK. Analysis of the interface surfaces and oligomer stability was done with the Protein Interfaces, Surfaces, and Assemblies web server (PISA (46)), and calculation of RMSDs of atomic positions was done with SUPERPOSE (38). The structural figures were generated with PyMOL (Schrodinger Molecular Graphics System, version 1.3, DeLano Scientific, San Carlos, CA).

### Size-exclusion chromatography and multiangle light scattering

First, SEC experiments were carried out on a Varian ProStar 215 solvent delivery system coupled to a Varian ProStar 320 UV-visible detector. Separation was performed using BioSep 5 μm SEC-s2000 145 Å, 300 × 4.6 mm LC column (Phenomenex, Torrance, CA, USA). 50 mm sodium phosphate buffer, pH 6.65, containing 150 mm NaCl was used as an eluent at a 0.5 ml/min flow rate. met–Evasin-4 was injected at 0.1 mg/ml concentration and followed by absorbance at λ = 280 nm.

In addition, SEC-MALS was used to determine the oligomeric state of Evasin-4 by injecting 100 μl of protein at 5.3 mg/ml onto a Superdex 75 10/300 gel filtration column (GE Healthcare) in 20 mm Tris, 100 mm NaCl, pH 7.3, at 17 °C. SEC was performed with online static light scattering (miniDAWN TREOS, Wyatt Technology) and differential refractive index determination (Shimadzu RID-10A) on a Shimadzu HPLC system. Protein concentration at elution and massed average molecular mass was determined using standard protocols in the ASTRA6 program (Wyatt). The set-up was calibrated with 100 μl of 5 mg/ml monomeric chicken egg white albumin (Sigma–Aldrich). A refractive index increment (*dn*/*dc*) value of 0.185 ml/g was used for Evasin-4.

### Surface plasmon resonance biosensor analysis

SPR biosensor analysis was performed using a BIAcore T200 surface plasmon resonance system (GE Healthcare). PBS buffer, pH 7.4, supplemented with 0.05% Tween 20 was used as running buffer at a flow rate of 30 µl/min. 40 response units of met–Evasin-4 randomly labeled with EZ-Link Sulfo-NHS-Biotin (ThermoFisher Scientific) were immobilized on a SAHC 200M sensorchip (Xantec) according to the manufacturer's protocol. Concentration series of chemokines, ranging from 7.8 nm to 2 μm, were prepared by 2-fold dilutions of the stock solution. Obtained sensograms were fitted by BIAEvaluation software (GE Healthcare), and apparent *K_D_* values were calculated using a steady-state affinity equation.

### Fluorescence anisotropy assay

Fluorescence polarization assays were carried out using FluoroMax 4 fluorimeter (Horiba) equipped with FM4-2000 polarizers in 3-ml cuvettes. 100 nm solution of 5(6)-Fam–labeled peptides in HEPES buffer pH 7.4 were titrated by a 30 μm stock solution of CCL5 at 37 °C. Fluorescent signal was measured using 495 and 520 nm as excitation and emission wavelengths. The final titration curve is a result of three or four independent measurements.

### THP-1 monocytic cell migration assay

Human acute monocytic leukemia cell line (THP-1) monocytic cells were purchased from Leibniz-Institut DSMZ (ACC 16). The cells were cultured in RPMI 1640 GlutaMAX medium supplemented with 20 % fetal calf serum and 1 % penicillin/streptomycin (all Gibco ThermoFisher). The cells were cultured in a humidified atmosphere (5 % CO_2_, 95 % air, 37 °C) and routinely passed by splitting the cells (1:10) to fresh medium. The migration of monocytic cells toward chemoattractants was assessed in a 12-well chemotaxis chamber equipped with a 5-μm polycarbonate membrane (both Neuroprobe, Gaithersburg, MD, USA). Chemoattractants CCL5 (0.5 µg/ml) or CXCL4 (0.5 µg/ml) were preincubated with met–Evasin-4 (1 µg/ml) for 2 h at 37 °C subsequently added to the bottom well of the chemotaxis chamber. THP-1 monocytic cells (1 × 10^6^/ml), suspended in RPMI 1640 GlutaMAX medium supplemented with 1 % fetal calf serum, were added in the upper wells and incubated for 90 min at 37 °C in a humidified atmosphere (5 % CO_2_, 95 % air). The nonmigrated cells were carefully removed, and the membrane was stained with Diff-Quick stain (Eberhard Lehmann GmbH, Berlin, Germany). Migrated cells were visualized by light microscopy, counted manually in five fields, and expressed as cells/mm^2^.

## Data availability

The structures presented in this paper have all been deposited in the PDB with the following codes: 6STK, 6ST4, 6STC, and 6STE. All remaining data are contained within the article.

## Supplementary Material

Supporting Information
