# Supplementary material for: Structural characterization of anti-CCL5 activity of the tick salivary protein evasin-4
Source: J Biol Chem. 2020 Aug 14;295(42):14367–78. doi: 10.1074/jbc.RA120.013891 (PMC7573271; doi:10.1074/jbc.RA120.013891)
Supplement: Supporting Information [file supp_295_42_14367__index.html]

Structural characterization of anti-CCL5 activity of the tick salivary protein Evasin-4 — Structural characterization of the tick protein Evasin-4 — Structural characterization of anti-CCL5 activity of the tick salivary protein evasin-4 — Structural characterization of the tick protein evasin-4 — Supporting Information 

# Structural characterization of anti-CCL5 activity of the tick salivary protein evasin-4

## Supporting Information

- Supporting Information (to be published online) - Detailed experimental procedures, NMR spectra, HPLC, etc
