# Supplementary material for: Structural characterization of anti-CCL5 activity of the tick salivary protein evasin-4
Source: J Biol Chem. 2020 Aug 14;295(42):14367–78. doi: 10.1074/jbc.RA120.013891 (PMC7573271; doi:10.1074/jbc.RA120.013891)
Supplement: Supporting Information [file supp_RA120.013891_160179_2_supp_581025_qf0143.pdf]

Supporting information

Structural characterization of anti-CCL5 activity of the tick salivary protein Evasin-4

Stepan S. Denisov[1*], Mercedes Ramírez-Escudero[2*], Alexandra C.A. Heinzmann[1], Johannes H. Ippel[1], Philip E. Dawson[3], Rory R. Koenen[1], Tilman M. Hackeng[1], Bert J.C. Janssen[2#] and Ingrid Dijkgraaf[1#]

[1]Department of Biochemistry, Cardiovascular Research Institute Maastricht (CARIM), Maastricht University, Universiteitssingel 50, 6229 ER, Maastricht, The Netherlands

[2]Crystal and Structural Chemistry, Bijvoet Center for Biomolecular Research, Faculty of Science, Utrecht University, 3584 CH Utrecht, The Netherlands

[3]Department of Chemistry, The Scripps Research Institute, 10550 N. Torrey Pines Road, La Jolla, California 92037, United States

* Authors contributed equally to the manuscript

[#] Corresponding authors. Email: b.j.c.janssen@uu.nl and i.dijkgraaf@maastrichtuniversity.nl

Table of content

Protein expression. S2

Evasin-4 folding and purification. S2

E66S CCL5 folding and purification. S2

[NMe-^{7}T] CCL5 synthesis and folding. S3

Synthesis of N-terminal peptides. S3

References S3

Table S1. Gene sequences used for expression S4

Table S2. Crystallographic data collection and refinement statistics S5

Table S3. HADDOCK statistics for the E66S CCL5/Ev4 E14-N31 complex. S6

Figure S1. Recombinant expression and folding met-Evasin-4. ..... S7

Figure S2. Recombinant expression and folding of 6His-SUMO-Evasin-4. ..... S8

Figure S3. NMR analysis of Evasin-4. ..... S9

Figure S5. Native chemical ligation of [NMe-^{7}T] CCL5. ..... S11

Figure S6. Folding of [NMe-^{7}T] CCL5. ..... S12

Figure S7. Mutation E66S does not disturb the CCL5 dimer architecture. S13

Figure S8. Synthesis of Ev4 E14-N31. ..... S14

Figure S9. Synthesis of Ev4 E14-A23. ..... S15

Figure S10. NMR analysis of [^{15}N, ^{13}C] E66S CCL5 monomer/dimer equilibria. S16

Figure S11. NMR analysis of Evasin-4 binding to [^{15}N, ^{13}C] E66S CCL5 ..... S17

Protein expression.

Recombinant met-CXCL4 was obtained from Dr. R. Koenen. The met-Evasin-4 pET30a, His6-SUMO-Evasin-4 pET23a and E66S MKKKWPR-CCL5 pET24a vectors (Table S1) were purchased from GenScript, USA and transfected to BL21 (DE3) Star cells (Novagen). Single colonies were inoculated overnight in standard LB media at 37°C in the presence of 100 mg/L of ampicillin or 50 mg/L of kanamycin. Overnight cultures were diluted 1:200 by fresh LB media and incubated at 37°C. Once the OD_{600} value reached 0.6-0.8, bacteria were induced with 0.1 mM or 1 mM of isopropyl β-D-1-thiogalactopyranoside (IPTG) (Sigma-Aldrich). Cells were harvested 3 h after induction by centrifugation at 4,000 rpm for 20 min at 4°C.

In order to obtain uniformly ^{13}C, ^{15}N-labeled proteins, bacteria containing met-Evasin-4 pET30a or E66S MKKKWPR-CCL5 CCL5 pET24a vector were grown in LB media as described above. After OD_{600} reached 0.6-0.8, cells were harvested by centrifugation at 4,000 rpm at 4°C and transferred to M9 media. Prior to induction with IPTG, cells were incubated in media for 1 h to deplete internal carbon and nitrogen sources. At induction, media was supplemented with 1 g/L of ^{13}C-glucose and $^{15}NH_4Cl$. To achieve a higher level of stable isotope enrichment, the concentration of ^{13}C-glucose was measured using a domestic glucosemeter (Medisana). Typically, after 3-4 h about 80% of glucose was consumed and cells were harvested by centrifugation at 4,000 rpm for 20 min at 4°C. For ^{2}D, ^{13}C, ^{15}N-labeled met-Evasin-4, M9 media containing 70% D_2O was used.

Evasin-4 folding and purification.

For lysis, met-Evasin-4 pET30a and His6-SUMO-Evasin-4 pET23a BL21 (DE3) Star pellets were resuspended in 6 M guanidine chloride (Gdn-HCl), 50 mM Tris, pH 8 at concentration of 0.2 mg/ml and stirred for 20 min in the presence of 1 mg/ml of lysozyme. Then, cell debris was removed by centrifugation at 10,000 rpm for 20 min at 4°C.

In the case of met-Evasin-4 pET30a pellets, the soluble fraction was dialyzed overnight against 0.5% acetic acid using a 3.5 kDa Spectra/Por RC membrane (Replige) and subsequently lyophilized. The lyophilized material was dissolved in a minimal volume of 6 M Gdn-HCl, 50 Mm Tris, pH 8, and diluted with 0.1 M Tris, 0.5 M NaCl, 1 mM EDTA, 2 mM GSH, 1 mM GSSG, 10% acetonitrile, pH 8 to a final concentration of 1 mg/ml and stirred overnight at 4°C.

The cleared lysate of His6-SUMO-Evasin-4 pET23a pellets was supplemented with 20 mM imidazole, applied to Ni-NTA Agarose (Quagen) and stirred for 1 h at RT. The suspension was transferred to an empty PD10 column and filtered under mild vacuum. Then, Ni-NTA Agarose was washed with one column volume of lysis buffer. Bound proteins were eluted by one column volume of 20 mM phosphate, 0.5 M NaCl, 0.5 M imidazole, pH 7.4. Eluted fractions were dialyzed against PBS buffer, pH 7.4. In order to remove the His6-SUMO tag, 5 U/ml of SUMO protease 1 (Sigma-Aldrich) and 0.6 mg of GSH were added and the resulting solution was gently stirred at 30°C. After cleavage completion, the solution was diluted 4 times by 0.1 M Tris, 0.5 M NaCl, 1 mM EDTA, 2 mM GSH, 1 mM GSSG, 10% acetonitrile, pH 8 and stirred overnight at 4°C. The folded proteins were purified by HPLC using a 22 mm x 250 mm Vydac C18 column, analyzed by LC-MS and lyophilized.

E66S CCL5 folding and purification.

Pellets were resuspended in 50 mM Tris, 2 mM $MgSO_4$, pH 8 and lysed with 1x Bugbuster (Novagen) and 0.1 U/ml benzonaze® nuclease (Sigma-Aldrich). The insoluble fraction was removed by centrifugation at 10,000 rpm for 20 min at 4°C, washed with 50 mM, 0.5% Tween-20, Tris, pH 8 and twice with 50 mM Tris, pH 8. Then, the insoluble fraction was dissolved in 0.1 M Tris, 200 mM DTT, pH 8, 6 M Gdn-HCl and dialyzed overnight against 0.5% acetic acid using 3.5 kDa Spectra/Por RC membrane (Repligen) and lyophilized. Lyophilized material was then dissolved in a minimal volume of 6 M Gdn-HCl, 50 mM Tris, pH 8, then diluted with 6 M Gdn-HCl, 50 Mm Tris, 10 mM cysteine, 1mM cystine, pH 8 to a final concentration of 1 mg/ml and stirred overnight at 4°C. The folded protein was purified by HPLC using a 22 mm x 250 mm Vydac C18 column, analyzed by LC-MS and

lyophilized. In order to remove MKKKWPR- tag, the purified protein was dissolved in 100 mM ammonium bicarbonate buffer pH 8.5 and treated with trypsin (Sigma-Aldrich). After cleavage completion, E66S CCL5 was purified by HPLC and lyophilized.

[NMe-^{7}T] CCL5 synthesis and folding.

The obligatory CCL5 monomer which contains a methylated amide nitrogen of T7 ([NMe-^{7}T] CCL5) (1) was synthesized using Boc-based solid-phase peptide synthesis (SPPS) and native chemical ligation (NCL). In short, the N-terminal NH_2-Ser^{1}-Lys^{33}-MPAL-COOH and C-terminal NH_2-Cys^{34}-Ser^{68}-$CONH_2$ segment were synthesized on Boc-Leu-PAM and MBHA resin, respectively. After chain assembly, the peptides were deprotected and cleaved from the resin by treatment with anhydrous hydrogen fluoride (HF) for 1 h at 0°C, using 4% (v/v) *p*-cresol as a scavenger. Crude peptides were lyophilized and purified by HPLC as described above. To allow NCL, unprotected peptides (10 mg/ml) were dissolved in 6 M Gdn-HCl, 100 mM Tris buffer, pH 8, containing 1% (v/v) benzylmercaptan and thiophenol and incubated at 37°C. Reaction progress was analyzed by analytical HPLC and ESI/MS or with UPLC-MS (Fig. S7). Ligated material was purified by HPLC, lyophilized and subjected to oxidative folding. Oxidative folding of the proteins was performed at 4°C in 1 M Gdn-HCl, 100 mM Tris buffer, pH 8.0, containing 1 mM cystine/10 mM cysteine as a redox couple. After folding completion, [NMe-^{7}T] CCL5 was purified by HPLC and lyophilized (Fig. S8).

Synthesis of N-terminal peptides.

Ev4 E14-N31 (H_2N-EEEDDYTAYAPLTAYFTN-$CONH_2$) and Ev4 E14-A23 (H_2N-EEEDDYTAYA-$CONH_2$) were synthesized by Boc-SPPS using MBHA resin and purified by preparative HPLC as described previously.

For labeling with a fluorescent probe, Boc-Lys(Fmoc)-OH was coupled to Ev4 E14-N31 and Ev4 E14-A23 peptidyl resin. The fluorenylmethoxycarbonyl (Fmoc) protecting group was removed by treatment by 20% piperidine in DMF. Subsequently, excess of 5-(and-6)-Carboxyfluorescein, Succinimidyl Ester (5(6)-Fam, SE) in DMF was added to the peptidyl resin in the presence of DIPEA and incubated overnight at 37°C and constant shaking. Then the peptidyl resin was deprotected by TFA and peptides were cleaved from resin by HF treatment. Labeling yielded two isomers with the ratio close to 1:1. Isomers were purified, pooled together and used as a mixture.

References

1. Proudfoot, A. E. I., Handel, T. M., Johnson, Z., Lau, E. K., LiWang, P., Clark-Lewis, I., Borlat, F., Wells, T. N. C., and Kosco-Vilbois, M. H. (2003) Glycosaminoglycan binding and oligomerization are essential for the in vivo activity of certain chemokines. *Proc. Natl. Acad. Sci. U. S. A.* **100**, 1885–1890

Table S1. Gene sequences used for expression.

Vector name	ORF
met-Evasin-4<br>pET30a<br>*NdeI-EcoRI*	catatggaagtaccccaaatgacttcaagttcagctccagatctagaagaagaagatgat<br>H M E V P Q M T S S S A P D L E E E D D<br>tacaccgcgtatgcgccgctgacctgctacttcaccaacagcaccctgggtctgctggct<br>Y T A Y A P L T C Y F T N S T L G L L A<br>ccgccgaactgcagcgtgctgtgcaacagcaccaccacctggttcaacgagaccagcccg<br>P P N C S V L C N S T T T W F N E T S P<br>aacaacgcgagctgcctgctgaccgttgactttctgacccaggatgcgatcctgcaggaa<br>N N A S C L L T V D F L T Q D A I L Q E<br>aaccaaccgtacaactgcagcgttggtcactgcgacaatggcacctgcgcgggtccgccg<br>N Q P Y N C S V G H C D N G T C A G P P<br>cgtcatgcgcaatgctggtaagaattc<br>R H A Q C W - E F
His6-SUMO-<br>Evasin-4<br>pET23a<br>*NdeI-EcoRI*	catatgggtcatcatcatcaccaccacggcagcctgcaagatagcgaagtgaaccaagaa<br>H M G H H H H H H G S L Q D S E V N Q E<br>gcgaaaccggaagttaaaccggaagtgaaaccggaaacccacatcaacctgaaggtgagc<br>A K P E V K P E V K P E T H I N L K V S<br>gatggtagcagcgagatcttctttaaaattaagaaaaccaccccgctgcgtcgtctgatg<br>D G S S E I F F K I K K T T P L R R L M<br>gaagcgttcgcgaagcgtcagggtaaagagatggacagcctgcgttttctgtacgatggc<br>E A F A K R Q G K E M D S L R F L Y D G<br>atccgtattcaggcggaccaagcgccggaagacctggatatggaggacaacgatatcatt<br>I R I Q A D Q A P E D L D M E D N D I I<br>gaagcgcaccgtgagcagattggtggcgaagttccgcaaatgaccagcagcagcgcgccg<br>E A H R E Q I G G E V P Q M T S S S A P<br>gacctggaggaagaggacgattacaccgcgtatgcgccgctgacctgctacttcaccaac<br>D L E E E D D Y T A Y A P L T C Y F T N<br>agcaccctgggcctgctggctccgccgaactgcagcgtgctgtgcaacagcaccaccacc<br>S T L G L L A P P N C S V L C N S T T T<br>tggttcaacgaaaccagcccgaacaacgcgagctgcctgctgaccgttgactttctgacc<br>W F N E T S P N N A S C L L T V D F L T<br>caagatgcgatcctgcaggagaaccaaccgtataactgcagcgtgggtcactgcgataat<br>Q D A I L Q E N Q P Y N C S V G H C D N<br>ggcacctgcgcgggtccgccgcgtcatgcgcaatgctggtaagaattc<br>G T C A G P P R H A Q C W - E F
E66S<br>MKKKWPR-<br>CCL5 pET24a<br>*NdeI-BamHI*	catatgaagaaaaaatggccgcgttctccgtatagctctgataccacgccgtgctgtttt<br>H M K K K W P R S P Y S S D T T P C C F<br>gcctatattgcacgtccgctgccgcgtgcccatatcaaagaatattttacaccagtggc<br>A Y I A R P L P R A H I K E Y F Y T S G<br>aaatgcagcaacccggcagtggttttcgtgacgcgtaaaaaccgccaggtttgtgcgaat<br>K C S N P A V V F V T R K N R Q V C A N<br>ccggagaaaaaatgggtgcgcgaatatattaatagtctgagcatgtcttaaggatc<br>P E K K W V R E Y I N S L S M S - G

Table S2. Crystallographic data collection and refinement statistics.

Crystal data	E66S CCL5 (PDB 6STK)	Evasin-4 crystal form 1 (PDB 6ST4)		Evasin-4 crystal form 2 (PDB 6STC)	Evasin-4 crystal form 3 (PDB 6STE)
		Native	Anomalous		
**Unit cell parameters**					
a, b, c (Å)	23.9, 56.3, 94.0	68.0, 68.0, 41.6	68.0, 68.0, 41.6	71.1, 71.1, 43.1	68.2, 68.2, 181.5
α, β, γ (°)	90, 90, 90	90, 90, 120	90, 90, 120	90, 90, 120	90, 90, 120
Space group	$P2_12_12_1$	$P3_221$	$P3_221$	$P3_221$	$P3_221$
**Data collection**					
Beamline	DLS (I24)	ESRF (ID29)	in-house	ESRF (ID30-A3)	ESRF (ID29)
Temperature (K)	100	100	100	100	100
Wavelength (Å)	0.9787	0.97717	1.5418	0.9677	0.97717
**Data processing**					
Resolution (Å)	48.31-1.52 (1.55-1.52)	58.89-1.29 (1.32-1.29)	28.89-2.00 (2.14-2.00)	43.06-1.69 (1.72-1.69)	59.07-1.79 (1.85-1.79)
Unique reflections	20411 (972)	28245 (2050)	7738 (1385)	14083 (722)	47147 (3398)
Multiplicity	7.4 (7.5)	9.6 (9.7)	21.21 (10.0)	3.8 (4.0)	19.4 (19.9)
Completeness (%)	100 (100)	100 (100)	99.96 (99.9)	98.3 (100)	99.97 (99.85)
Mean $I/\sigma(I)$	13.8 (1.5)	8.6 (2.1)	40.11 (10.7)	11.7 (1.4)	7.1 (3.0)
R_{merge} (%)	6.9 (143.9)	14.3 (92.9)	5.98 (15.11) *	5.4 (92.8)	26.0 (101.8)
R_{pim} (%)	2.7 (55.9)	4.9 (31.4)	3.58 (10.53)	3.1 (53.2)	6.0 (23.3)
CC 1/2 (%)	99.9 (67.9)	98.5 (55.2)	n.d.	99.9 (49.6)	98.8 (55.9)
Molecules per ASU	2	1	1	1	4
Solvent content (%)	41.64	58.02	58.02	62.89	61.79
**Refinement**					
R_{work} / R_{free} (%)	18.04/20.28	17.81/19.43		17.58 / 19.89	17.93/20.91
**N° of atoms / average B** (Å^2)					
Protein	1048 / 29.77	669 / 31.81		687 / 33.96	2513 / 38.17
Waters	136 / 38.15	65 / 40.38		49 / 44.11	238 / 46.38
Solutes	10 / 59.21	24 / 42.38		33 / 62.92	37 / 44.66
All atoms	1194 / 30.97	758 / 32.88		769 / 35.85	2788 / 38.83
**Ramachandran plot** (%)					
Favoured	100.00	95.29		100.00	98.12
Outliers	0	0		0	0
**RMS deviations**					
Bonds (Å)	0.009	0.015		0.014	0.015
Angles (°)	1.36	1.82		1.72	1.42

Values in parentheses are for the high-resolution shell, * indicates R anomalous, n.d. not determined

Table S3. HADDOCK statistics for the E66S CCL5/Ev4 E14-N31 complex.

HADDOCK score	92.2 +/- 2.0
Cluster size	18
RMSD from the overall lowest-energy structure	16.0 +/- 0.1
Van der Waals energy	-56.5 +/- 6.5
Electrostatic energy	-40.4 +/- 8.6
Desolvation energy	-32.4 +/- 3.1
Restraints violation energy	47.9 +/- 12.76
Buried Surface Area	1478.5 +/- 73.7
Z-Score	-1.4

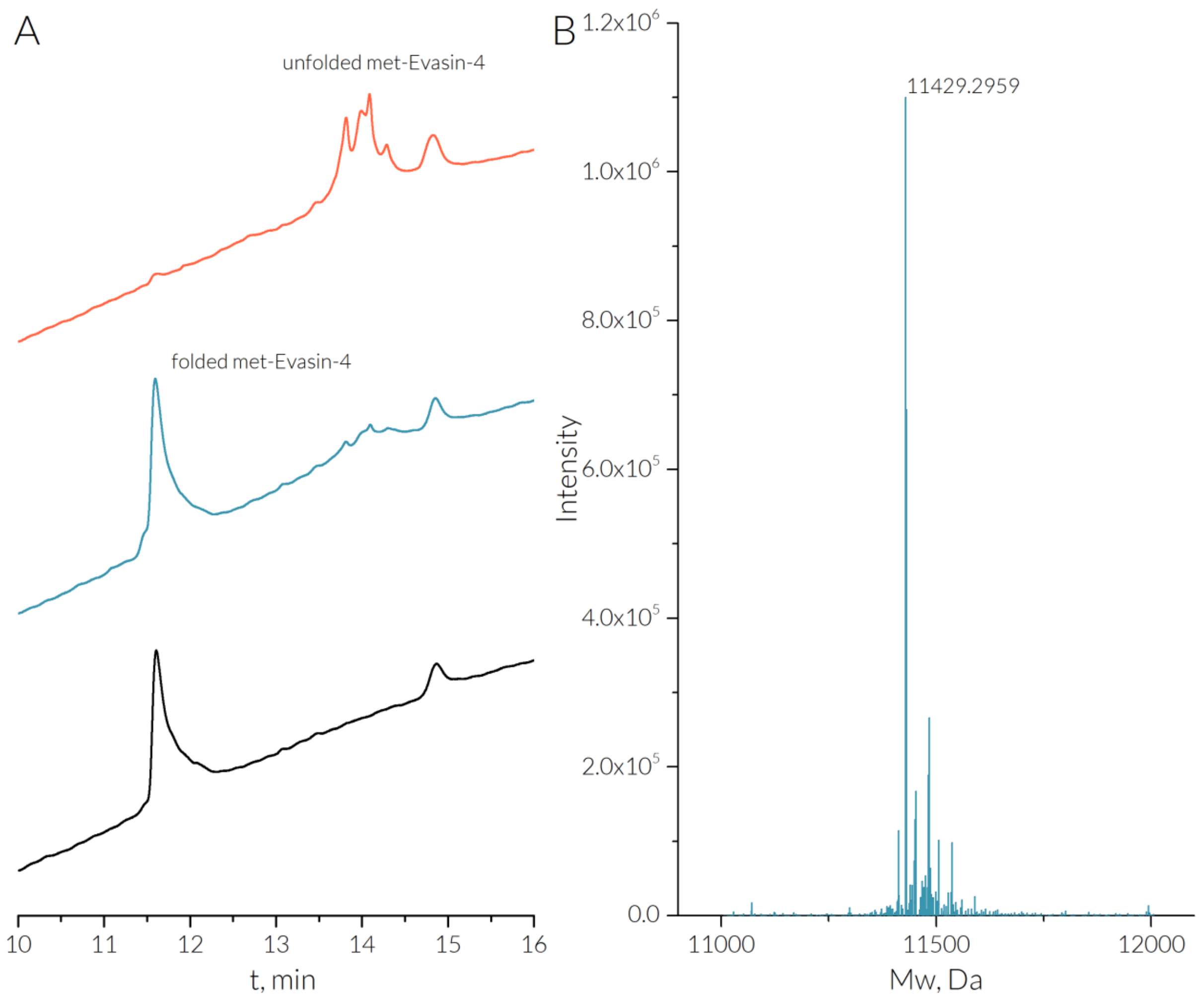

Figure S1. Recombinant expression and folding met-Evasin-4. A. HPLC traces of crude lysate of met-Evasin-4 from top to bottom: before oxidative folding; after oxidative folding; and after purification. **B.** Deconvoluted mass spectrum extracted from LC-MS analysis of purified met-Evasin-4; calculated monoisotopic mass of met-Evasin-4 is 11429.00.

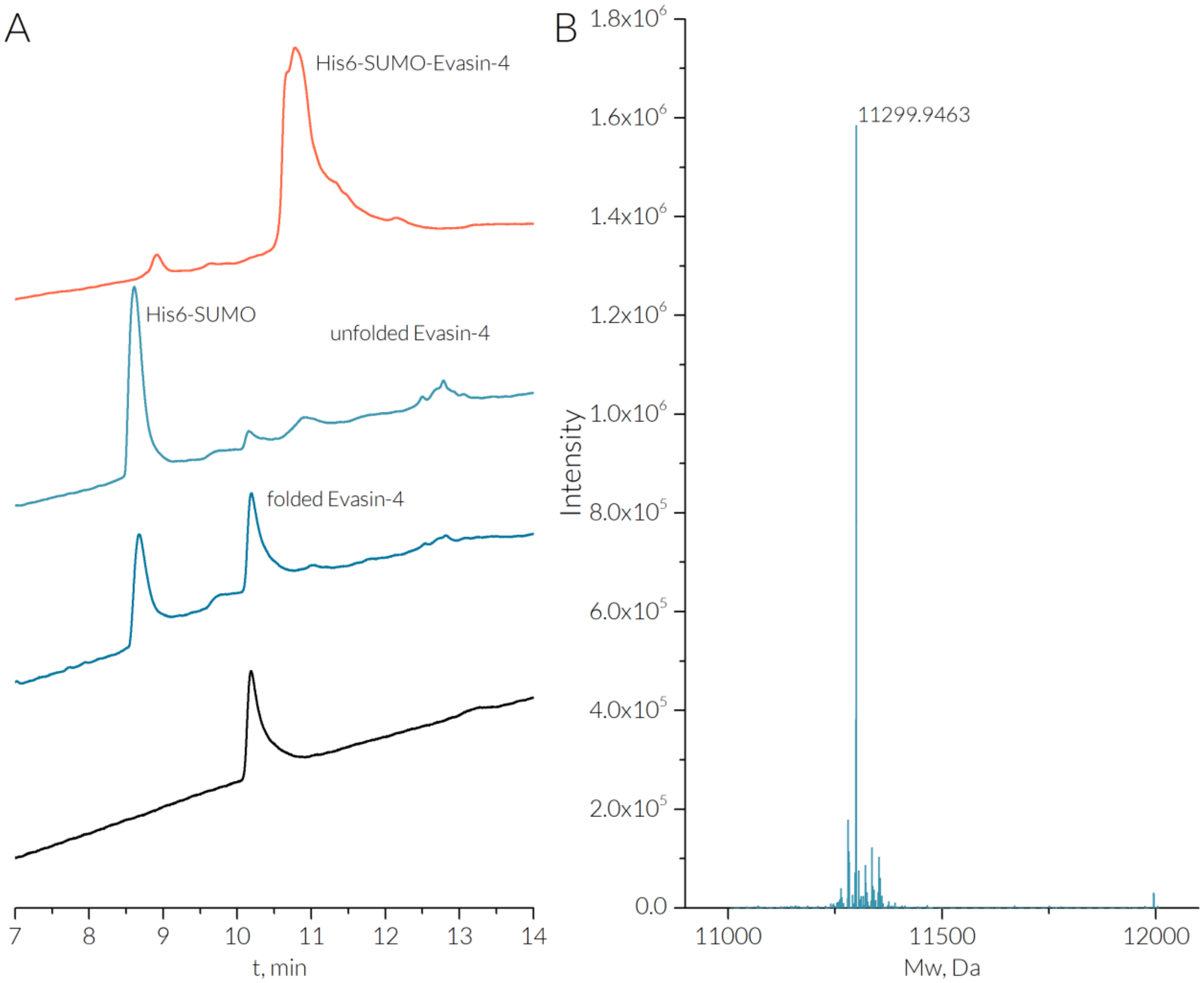

Figure S2. Recombinant expression and folding of 6His-SUMO-Evasin-4. A. HPLC traces of crude lysate of met-Evasin-4 from top to bottom: crude lysate after purification on Ni NTA Agarose; cleavage with SUMO protease; oxidative folding; and after purification. **B.** Deconvoluted mass spectrum extracted from LC-MS analysis of purified Evasin-4; calculated monoisotopic mass of Evasin-4 is 11297.96.

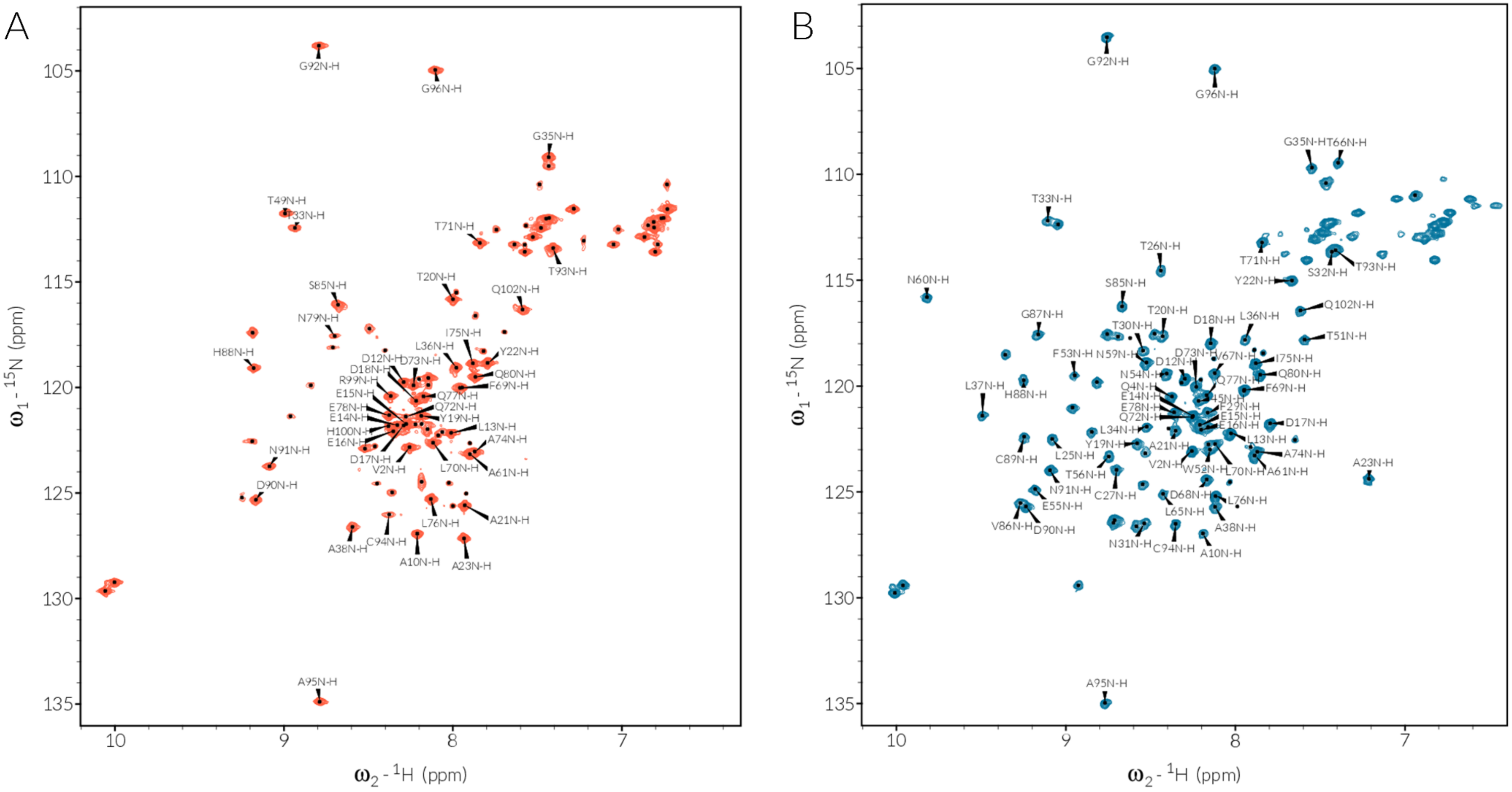

Figure S3. NMR analysis of Evasin-4. ^{15}N-^{1}H HSQC spectrum of 50 µM [^{15}N, ^{13}C] met-Evasin-4 at 37°C, pH 7.1 (**A**) and 100 µM E66S CCL5/[^{15}N, ^{13}C, ^{2}D] met-Evasin-4 complex at 37°C, pH 7.1 (**B**). Assignments of asparagine, glutamine, and arginine side chain peaks are left out for improved visibility.

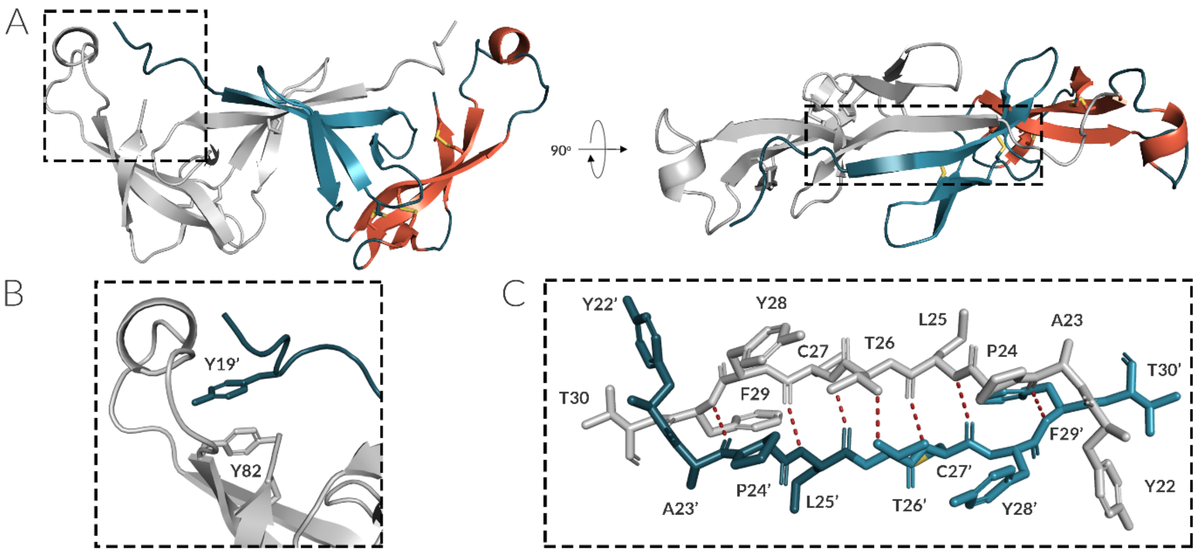

Figure S4. Evasin-4 has an intermolecular interface common in the three crystal forms. A. The front and top view of the cartoon representation of the Evasin-4 interactions *in crystallo* (representation of two asymmetrical units observed in the crystal form 6ST4). For visibility, one Evasin-4 molecule is colored as in Fig. 1 and the second molecule is shown in grey. **B-C.** Stick representation of ancillary (**B**) and the central intermolecular β-strands (**C**) interactions between Evasin-4 molecules (dashed boxes in panel **A**). The intermolecular interface includes 11 hydrogen bonds (indicated with dashed lines in **C**) between the main chain of the two strands β1 (Y22-T30), and between the strand β1 (E16-Y19) of one molecule and the loop β5-β6 (Q77-Y82) of the other molecule. In addition, several stacking and T-shaped hydrophobic interactions were also observed, namely interactions mediated by N-terminal residues (P24/Y28 and L25/ F29 interactions (**C**), and between Y19 with a hydrophobic or aromatic residue of the β5-β6 loop (**B**).

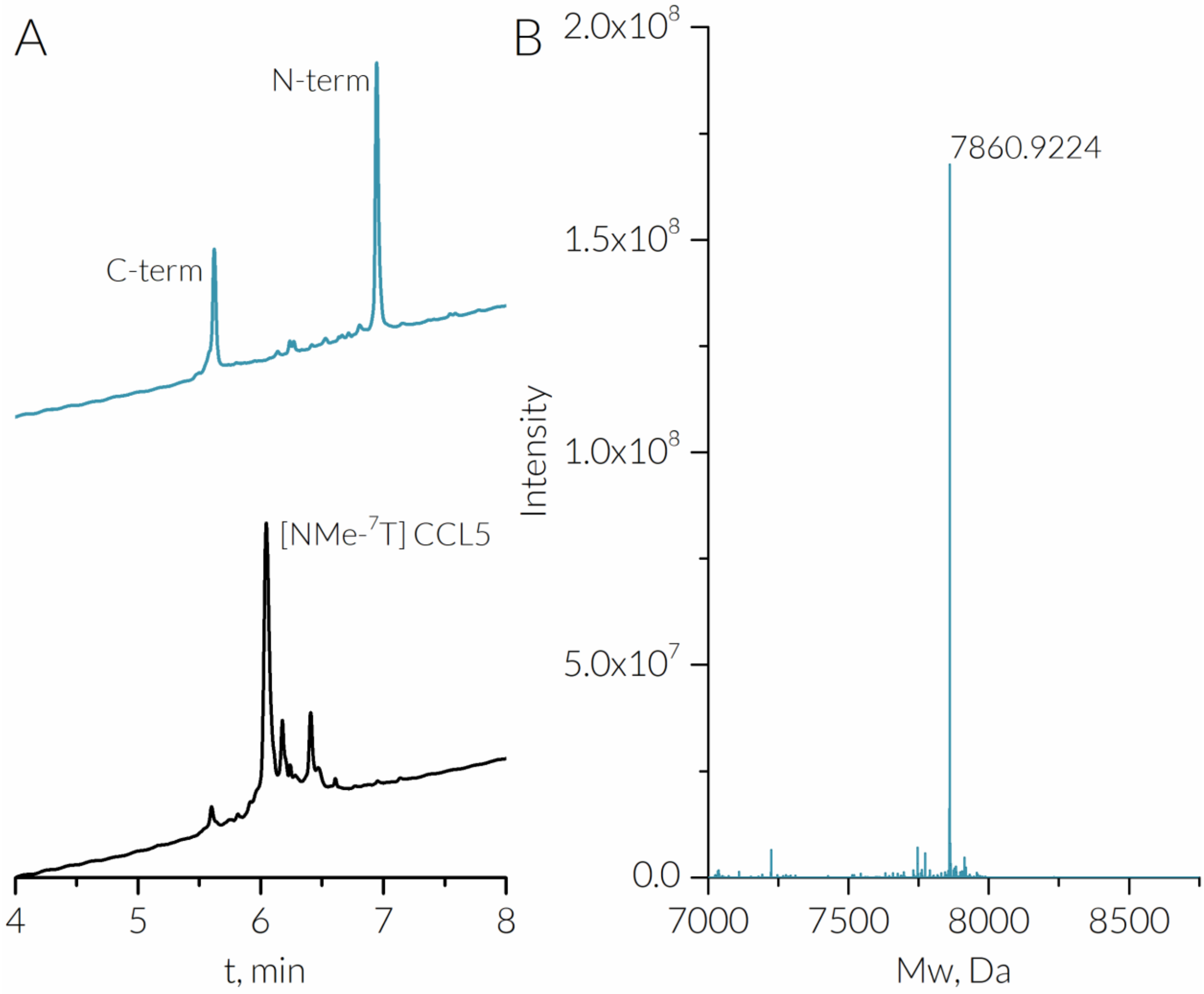

Figure S5. Native chemical ligation of [NMe-^{7}T] CCL5. A. HPLC traces of [NMe-^{7}T] CCL5 ligation at t = 0 (top) and t = 17 h (bottom). **B.** Deconvoluted mass spectrum extracted from LC-MS analysis of purified unfolded [NMe-^{7}T] CCL5; calculated monoisotopic mass of unfolded [NMe-^{7}T] CCL5 is 7859.83.

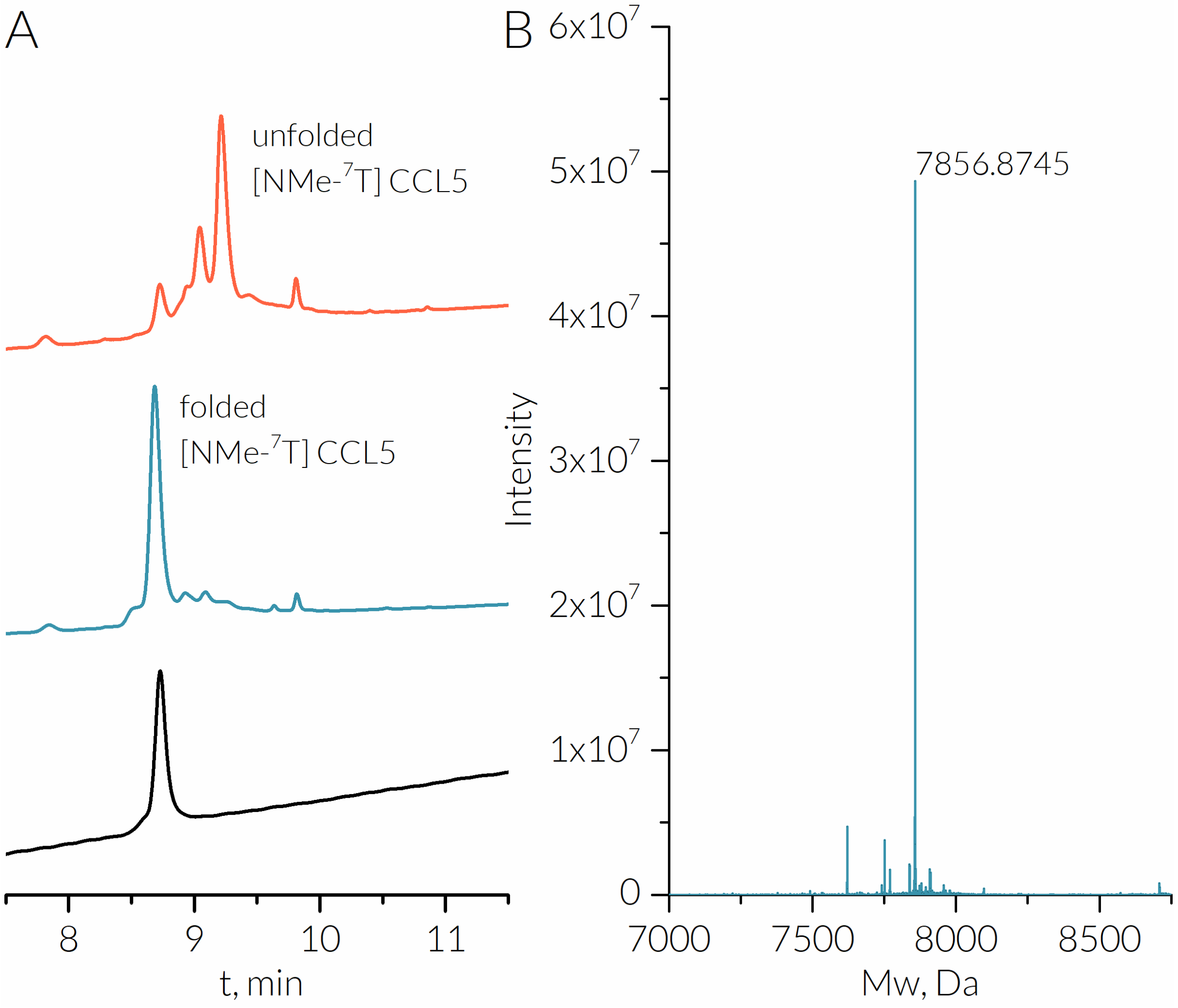

Figure S6. Folding of [NMe-^{7}T] CCL5. A. HPLC traces of [NMe-^{7}T] CCL5 folding from top to bottom: at t = 0; t = 3 h; and after purification. **B.** Deconvoluted mass spectrum extracted from LC-MS analysis of purified folded [NMe-^{7}T] CCL5; calculated monoisotopic mass of folded [NMe-^{7}T] CCL5 is 7855.83.

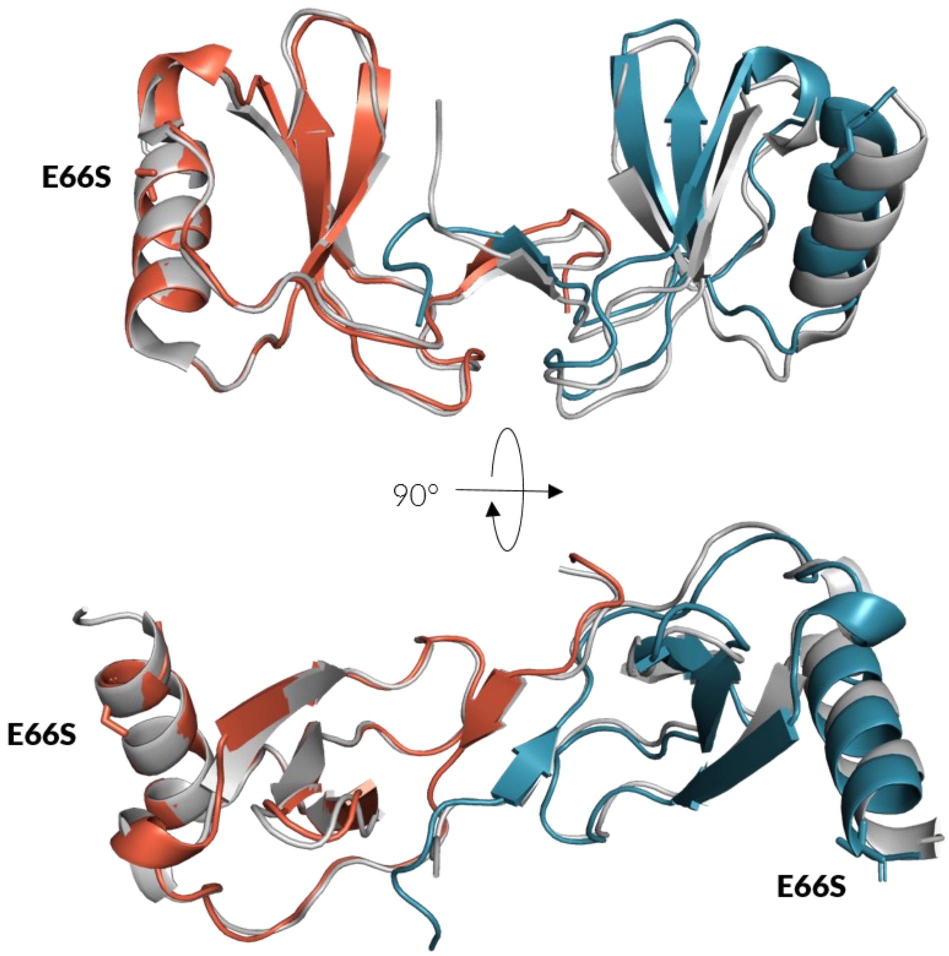

Figure S7. Mutation E66S does not disturb the CCL5 dimer architecture. Structural superposition of CCL5 wildtype (grey, PDB code 5COY) to CCL5-E66S mutant (orange and blue, PDB code 6STK). Mutation on residue 66 are highlighted in sticks.

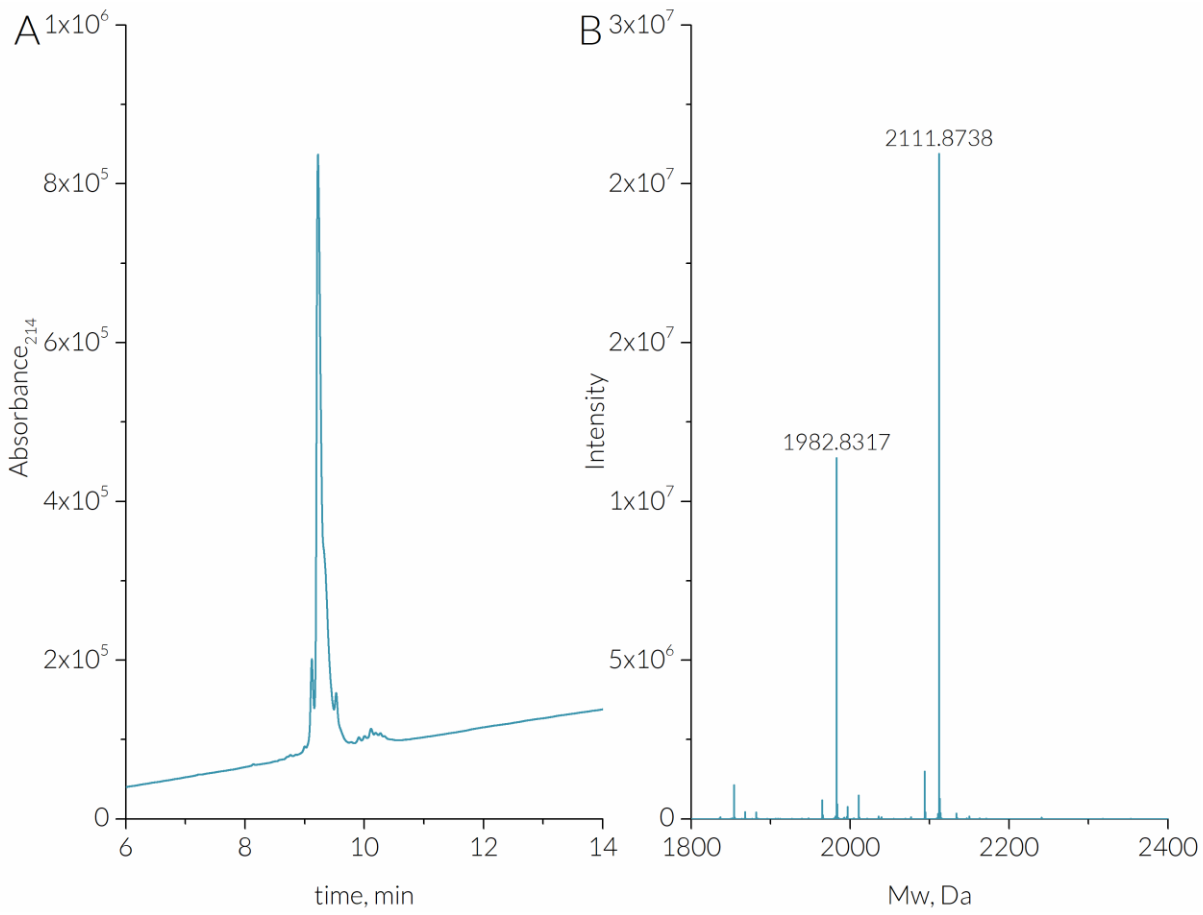

Figure S8. Synthesis of Ev4 E14-N31. A. HPLC traces of Ev4 E14-N31. **B.** The deconvoluted mass spectrum extracted from LC-MS analysis of purified Ev4 E14-N31; calculated monoisotopic mass of Ev4 E14-N31 is 2110.89. The second peak with the mass of 1982.83 represents a Glu deletion.

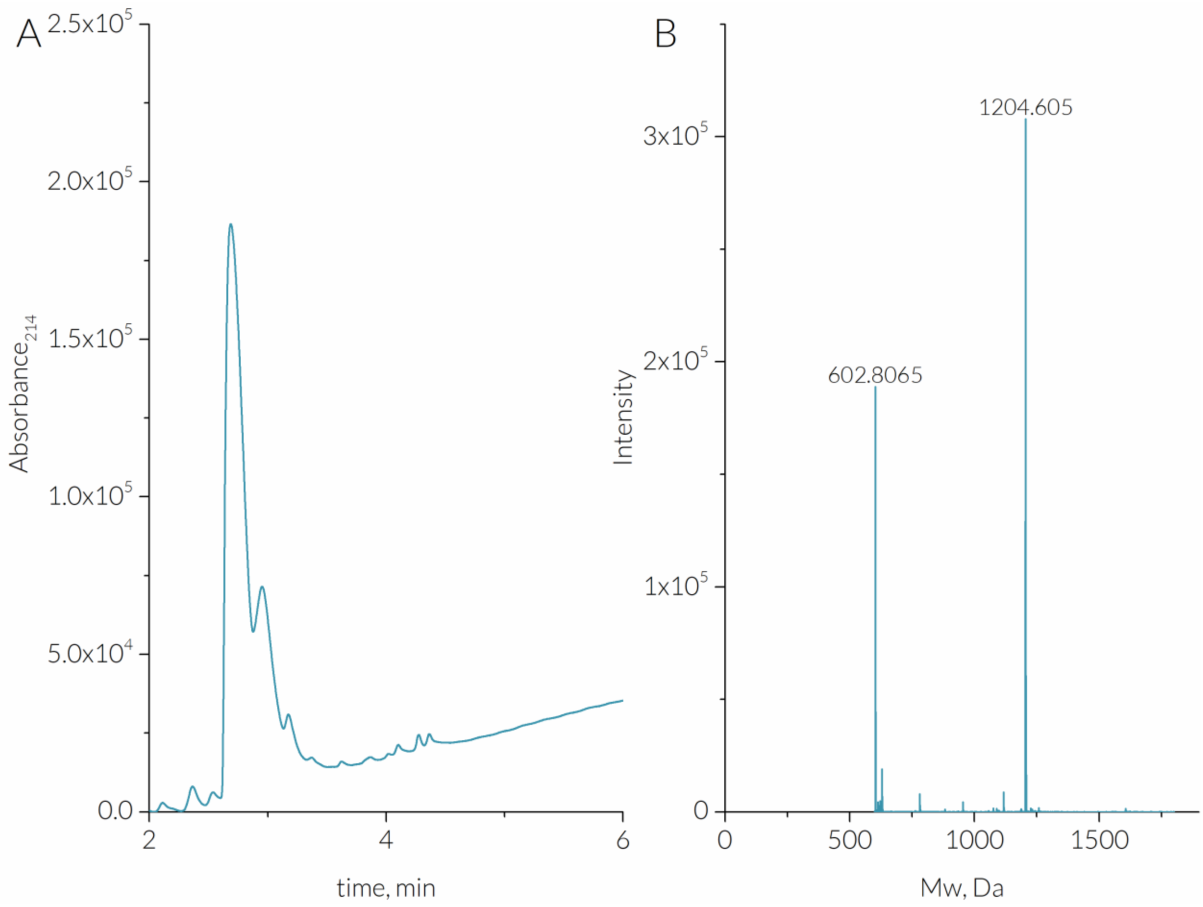

Figure S9. Synthesis of Ev4 E14-A23. A. HPLC traces of Ev4 E14-A23. The double peak arises due to possible isomerization. **B.** The mass spectrum extracted from LC-MS analysis of purified Ev4 E14-A23; calculated monoisotopic mass of Ev4 E14-A23 is 1203.44.

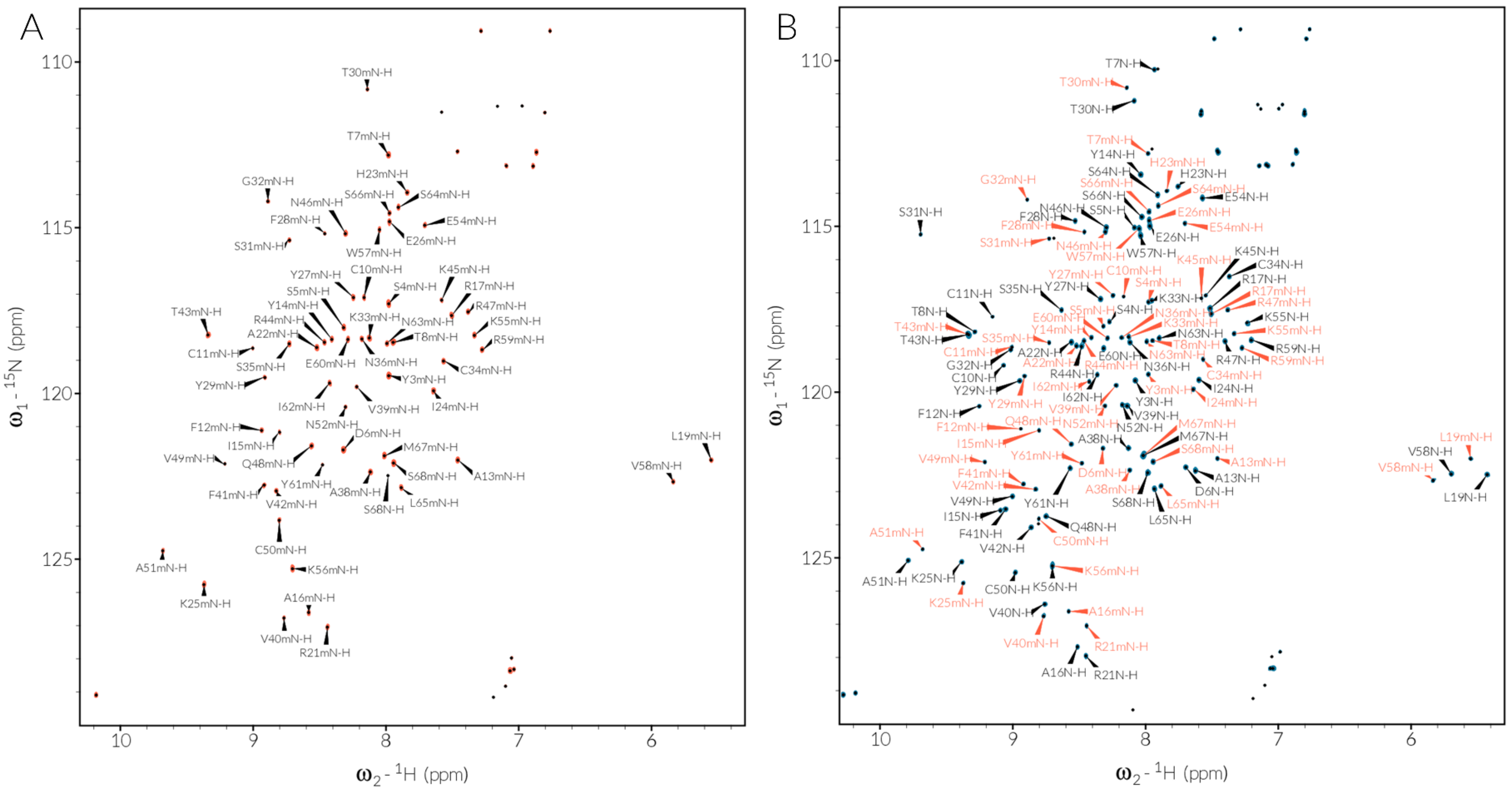

Figure S10. NMR analysis of [^{15}N, ^{13}C] E66S CCL5 monomer/dimer equilibria. ^{15}N-^{1}H HSQC spectrum of 1 μM (**A**) and 20 μM (**B**) [^{15}N, ^{13}C] E66S CCL5 at 37°C, pH 4. In the spectrum of 20 μM [^{15}N, ^{13}C] E66S CCL5 amide peaks of the monomeric form are labeled in orange; the dimeric form – in black. Assignments of asparagine, glutamine, and arginine side chain peaks are left out for improved visibility.

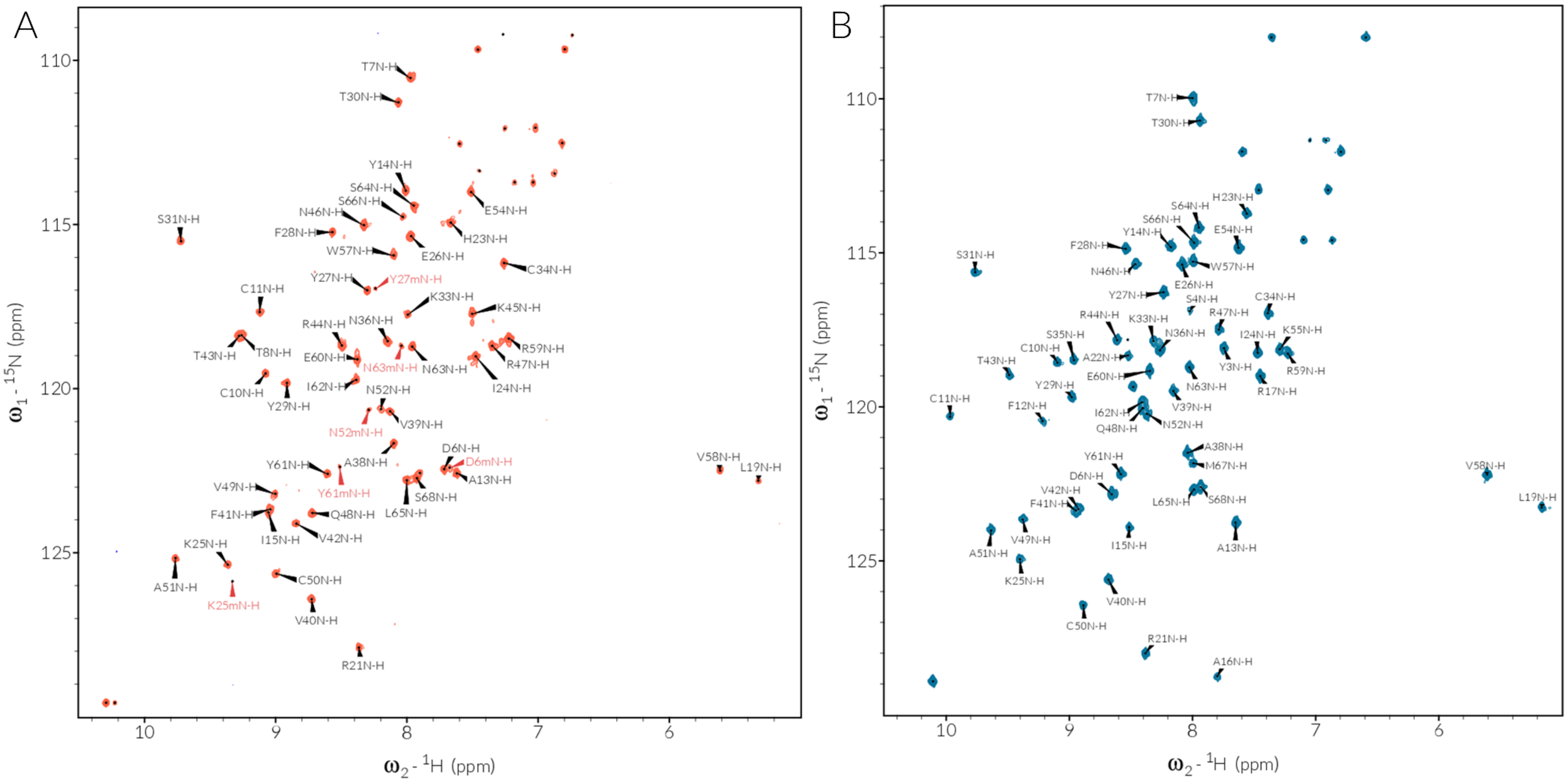

Figure S11. NMR analysis of Evasin-4 binding to [^{15}N, ^{13}C] E66S CCL5. ^{15}N-^{1}H HSQC spectrum of 20 µM [^{15}N, ^{13}C] E66S CCL5 at 37°C, pH 7.3 (**A**) and 80 µM [^{15}N, ^{13}C] E66S CCL5/met-Evasin-4 complex at 37°C, pH 7.0 (**B**). In the spectrum of free [^{15}N, ^{13}C] E66S CCL5 amide peaks of the monomeric form are labeled in orange; the dimeric form – in black. Assignments of asparagine, glutamine, and arginine side chain peaks are left out for improved visibility.
